# The dopamine circuit as a reward-taxis navigation system

**DOI:** 10.1371/journal.pcbi.1010340

**Published:** 2022-07-25

**Authors:** Omer Karin, Uri Alon

**Affiliations:** 1 Dept. of Molecular Cell Biology, Weizmann Institute of Science, Rehovot Israel; 2 Dept. of Applied Mathematics and Theoretical Physics, Centre for Mathematical Sciences, University of Cambridge, Cambridge, United Kingdom; 3 Wellcome Trust/Cancer Research UK Gurdon Institute, University of Cambridge, Cambridge, United Kingdom; McGill University, CANADA

## Abstract

Studying the brain circuits that control behavior is challenging, since in addition to their structural complexity there are continuous feedback interactions between actions and sensed inputs from the environment. It is therefore important to identify mathematical principles that can be used to develop testable hypotheses. In this study, we use ideas and concepts from systems biology to study the dopamine system, which controls learning, motivation, and movement. Using data from neuronal recordings in behavioral experiments, we developed a mathematical model for dopamine responses and the effect of dopamine on movement. We show that the dopamine system shares core functional analogies with bacterial chemotaxis. Just as chemotaxis robustly climbs chemical attractant gradients, the dopamine circuit performs ‘reward-taxis’ where the attractant is the expected value of reward. The reward-taxis mechanism provides a simple explanation for scale-invariant dopaminergic responses and for matching in free operant settings, and makes testable quantitative predictions. We propose that reward-taxis is a simple and robust navigation strategy that complements other, more goal-directed navigation mechanisms.

## Author summary

Research on certain circuits in simple organisms, such as bacterial chemotaxis, has enabled the formulation of mathematical design principles, leading to ever more precise experimental tests, catalyzing quantitative understanding. It would be important to map these principles to the far more complex case of a vertebrate behavioral circuit. Here, we provide such a mapping for the midbrain dopamine system, a key regulator of learning, motivation, and movement. We demonstrate a mathematical analogy between the regulation of dopamine and movement by rewards and the well-characterized bacterial chemotaxis system. We use the analogy to quantitively explain previously puzzling observations on the dopamine circuit, as well as classic empirical observations on operant behavior.

## Introduction

**Fig 1:**
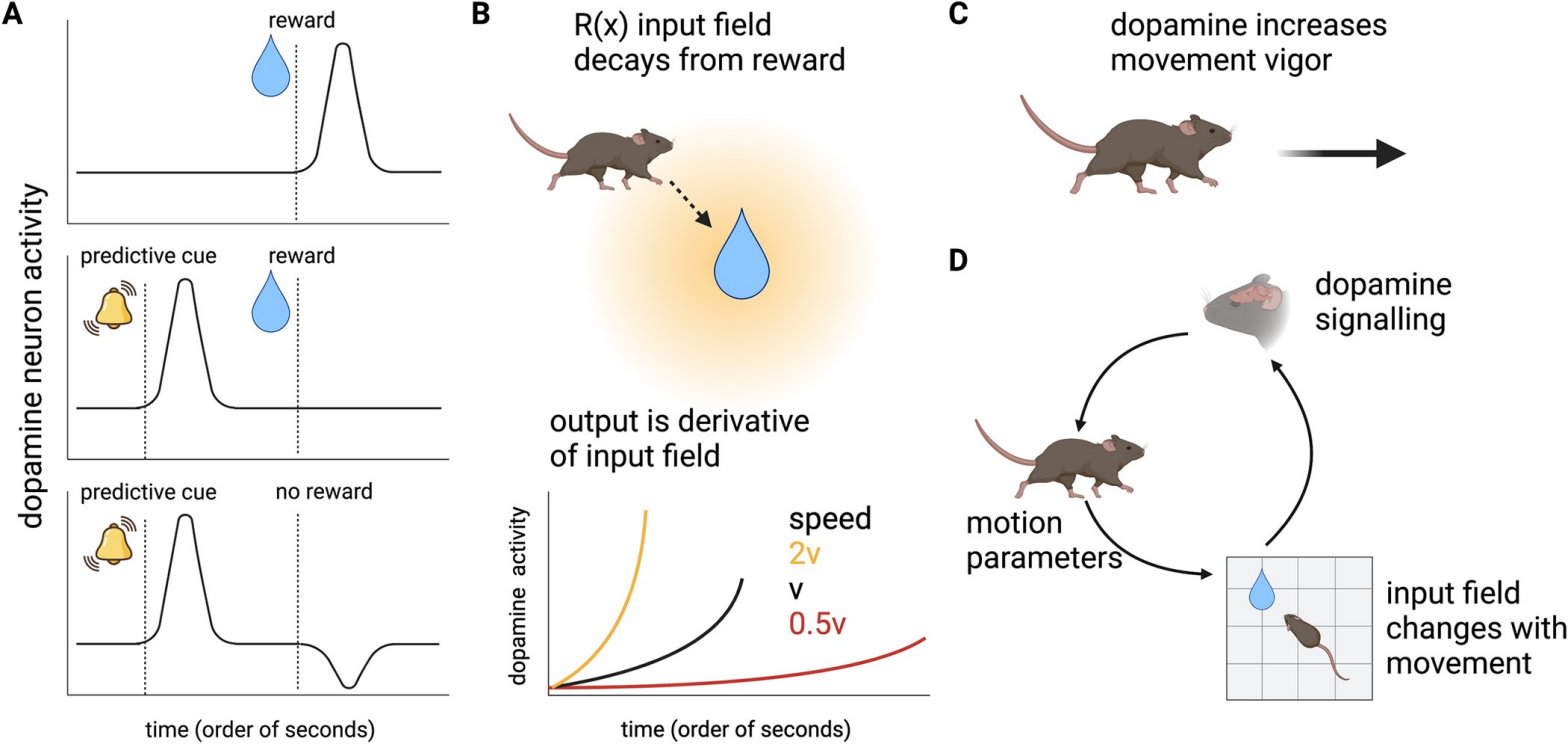
Experimental observations on dopamine imply feedback between motion and sensing. (A) Dopamine responses represent predictions errors over expected rewards, as observed in the classical experiments of Schultz [28]. Dopaminergic neurons fire at a constant rate of ~4-5Hz, and the delivery of a reward causes them to fire over this baseline rate (upper panel); however, when reward delivery is preceded by a predictive cue, the increase in firing occurs following the cue, and there is no increase following reward delivery (middle panel). Dopaminergic neurons fire below their baseline following the omission of an expected reward (lower panel). (B) As the animal approaches a reward, dopamine activity may increase, a phenomenon known as dopamine ramps. In elegant experiments based on virtual reality manipulations of animal movement (including change of movement velocity and teleportation), Kim et al. [12] demonstrated that dopamine ramps correspond to a temporal derivative calculation over a spatial input field that decays away from the reward. Thus, for example, movement at higher velocity leads to higher dopamine levels. (C) Finally, higher dopamine levels increase movement vigor. (D) There is thus feedback between dopamine sensing and movement–the movement of the animal (including movement velocity) changes the temporal derivative of the sensed input field, which affects dopamine levels, which then feed back on movement parameters. Figures were created with BioRender.com.

Dopamine transmission in the midbrain has several major biological functions for the regulation of behavior and learning. Dopamine signals encode *reward prediction errors (RPEs)* [1–6] (Fig 1A). Reward prediction errors are the difference between the experienced and predicted rewards. They play a key role in a method of reinforcement learning called temporal difference learning (TD learning) [5–8], and theory from reinforcement learning has been pivotal for explaining dopamine function.

In addition to rapid sub-second responses encoding reward prediction errors, dopamine may also slowly ramp up when approaching a reward [9–11]. A recent experiment by Kim et al. showed that even on this slower timescale (seconds), dopamine levels track a derivative of the input signal [12]. Kim et al. used virtual reality to manipulate the rate of change of the movement of a mouse as it moved towards a target. Dopamine changed in a way that is consistent with computing a temporal derivative of an input field that decays away from the reward (Fig 1B, such an input field is referred to as the spatially-discounted expected reward).

Finally, another well-established function of dopamine is the invigoration of movement and motivation [13–18]. Dopamine increases movement vigor [17] (Fig 1C) and defects in dopamine transmission underlie movement difficulties in Parkinson’s disease [19]. While the relation between RPEs and learning is well understood, it is unclear why an RPE signal should invigorate movement [18,20]. Theoretical studies have analyzed this question from the perspectives of learning [21,22] and cost-benefit theories [13,18,23,24], while early work on TD learning anticipated a connection with biological navigation [2].

Considering both the signal processing and movement-invigorating properties of dopamine reveals an intriguing feedback that is inherent to the system (Fig 1D). Since dopamine computes a temporal derivative on a spatial input field, the modulation of movement speed by dopamine should by itself affect dopaminergic output (as directly observed by Kim et al. [12], Fig 1C). Thus, in the context of a moving animal, the different roles of dopamine become tightly coupled. Analyzing such feedback interactions is challenging for current theoretical frameworks for dopamine, which typically model behavior using discrete choice processes occurring in discrete steps (see for example [25]).

Our study aims to use concepts from systems biology to analyze functional properties of the interaction between sensing and movement in the dopamine system. We first develop a minimal mechanistic model of dopamine responses. The model is similar to a continuous version of the classic TD-RPE model, with an important modification based on dopaminergic response curves–the circuit is activated by the logarithm of expected reward. Our model provides a new and simple explanation for the puzzling rescaling of dopaminergic responses [26]. Notably, the model establishes a connection between the dopamine circuit and the concepts of exact adaptation and fold-change detection, which have fundamental importance in the systems biology of signaling circuits [27].

We then use the model to study the interaction between dopamine signaling and movement. We considered one of the best-established empirical behavioral laws–the matching law of operant behavior, where the ratio of responses to concurrent rewarding alternatives is proportional to the ratio of obtained rewards raised to a power β (where β~1). Matching is typically observed in experiments where the animal is allowed to move freely, presenting a challenge to modelling approaches based on discrete choices and time steps. By considering a simple movement model, which we call *reward-taxis*, we show that the dopamine model predicts matching and provides a quantitative value for β as the ratio of dopamine gain to baseline activity. Matching results from the mathematical analogy between stochastic movement guided by *reward-taxis* and algorithms for the sampling of probability distributions. We conclude by proposing that *reward-taxis* is a simple and robust navigation strategy that complements other, more goal-directed navigation strategies employed by animals.

## Results

### Dopamine release as fold-change detection of expected reward

**Fig 2:**
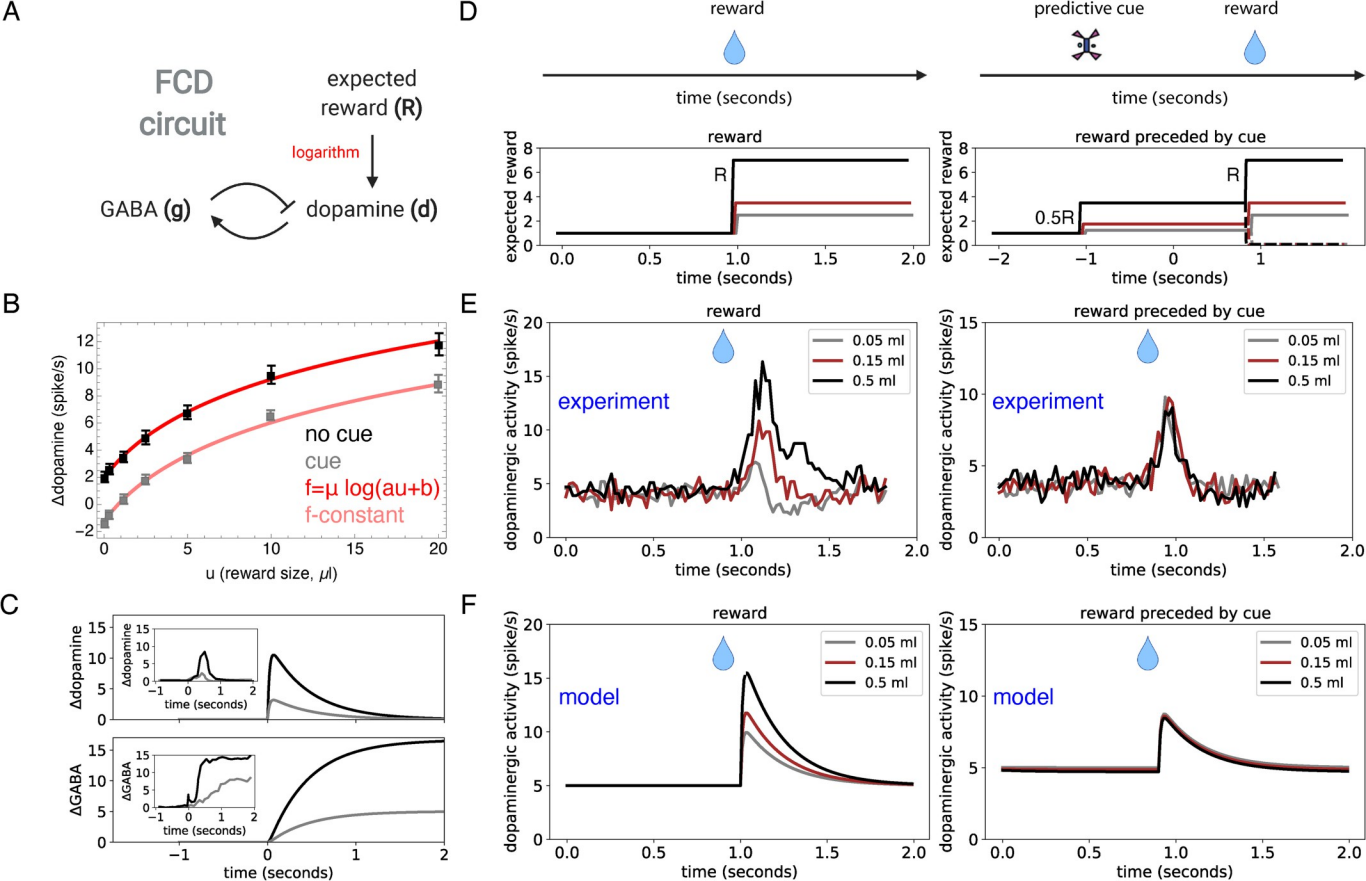
Logarithmic activation of dopamine dynamics explains scale invariant responses. (A) Minimal circuit for dopamine responses. Dopamine *(d)* is activated by the logarithm of expected reward (log *R*) and is inhibited by negative feedback from GABAergic input (*g*). See *Methods* for equations and parameters. (B) The response of VTA dopaminergic neurons in mice (n = 5) to a water reward of variable volume (black squares, mean ± SEM, taken from Fig 1C in [38]) is well-described by the logarithmic relation Δ*d* = *μ* log(*au*+*b*) (red line *r*^2^ = 0.999, best-fit parameters *a* = 0.5±0.1, *b* = 1.5±0.05, *μ* = 4.9±0.45, N = 7 reward magnitudes). When the reward is preceded by an odor cue (gray squares) the response is well-described by subtracting a constant from the uncued response (pink line, *r*^2^ = 0.99, best-fit subtraction constant is −3.2±0.1). (C) Simulation of dopamine and GABA responses to a step increase in expected reward input which corresponds to the presentation of a reward-predicting cue. The step is given by *R*(*t*) = *R*_0_+*λθ*(*t*−*t*_0_) where *θ*(*t*−*t*_0_) is a unit step function, *R*_0_ = 1 and *λ* = 7 for the black line (large reward) and *λ* = 1.8 for the gray line (small reward). *Insets*. Average change in firing rates from dopaminergic (type I) and GABAergic (type II) VTA neurons, in response to reward-predicting cues for a small reward (gray) or a large reward (black). Data from Fig 2D of [49]. (DE) Population responses of dopaminergic neurons of two Macaque monkeys to variable size liquid reward, either without a preceding cue (left panels, n = 55 neurons), or with a preceding visual cue that predicts reward delivery with 50% probability (right panels, n = 57 neurons). (D) The expected-reward input following the reward-predicting cue is *R* = 0.5(*b*+*λu*) (where *λ* is proportional to reward magnitude), which is doubled following reward delivery, *R* = *b*+*λu*, where *u* is the reward magnitude (we use *b* = 2 and *λ* = 10*ml*^−1^). Dashed lines correspond to reward omission. (E) Experimentally measured average dopaminergic responses, using data from Fig 2A and Fig 4B of [26]. When the reward is delivered without a cue, dopaminergic responses increase with reward magnitude (left panel). When it is given after a cue that predicts reward delivery with 50% probability, dopaminergic responses to reward delivery are identical (right panel), as predicted by the FCD property. This is despite the 10-fold difference in reward magnitude. (F) Simulations of the dopamine model capture the experimentally observed dynamics. All simulation parameters are provided in Table 1.

We begin by developing a minimal model for continuous dopamine dynamics (Fig 2A). Consider a behaving animal exploring an open field for a reward of magnitude *u*, such as a food or drink. For simplicity, we assume a uniform response amongst all dopaminergic neurons. In reality, there are heterogeneities between and within midbrain structures, and some dopaminergic neurons may be specialized to specific cues [29–31,17,32–35]. As the animal approaches the reward, there is an increase in expected reward *R*, which decays with distance from the target [9,12,36].

**Table 1:** Parameter values.

Parameter	Value (mouse experiments)	Value (primate experiments)
*ω*_*d*_	50 s^-1^	100 s^-1^
*ω*	15 s^-1^	30 s^-1^
*C*	15 spike s^-1^	15 spike s^-1^
*μ*	6 spike s^-1^	6 spike s^-1^
*α*	0.7	0.7
*d*_0_	5 spike s^-1^	5 spike s^-1^

Here expected reward is defined based on TD learning: the expected temporally discounted sum of present and future rewards (see Methods). According to the TD-RPE theory of dopamine function, the difference between dopamine and its baseline Δ*d* encodes a prediction error signal about expected rewards [4,5]. The prediction error signal allows the agent to learn about the spatial input field *R* with recursive learning rules [7,37].

What is the quantitative relationship between reward magnitude and the dopaminergic response? Recordings from VTA dopaminergic neurons in mice reveal a sublinear relationship between the magnitude of received rewards *u* and Δ*d*(*u*) [30,38] (Fig 2B). The sublinear relationship indicates that dopamine neurons may (at least in some magnitude range) be responding to the logarithm of reward, namely Δ*d*(*u*) = *μ* log(*R*(*u*)), with *R*(*u*) = *au*+*b*, where *a* is a scaling factor and *b* is a magnitude-independent component of the reward activation. The parameter *μ* is the gain of the dopaminergic response. Logarithmic responses are consistent with widespread logarithmic coding in the brain [39–44] as well as with economic notions of utility [45,46].

To test this, we fit the function *μ* log(*au*+*b*) to the average dopaminergic response to a variable water reward in mice [30], finding an excellent fit (*r*^2^ = 0.999), with a gain of *μ* = 4.94±0.45.

In addition to activation by expected reward, dopaminergic neurons in the VTA are inhibited following the presentation of a predicting cue in a subtractive manner ([30,38], Fig 2B, the presented cue is the same for all reward magnitudes). The subtractive inhibition is thought to be due to the increase in the activity of adjacent GABAergic neurons [30,38]. We therefore propose the following minimal description of dopamine release dynamics:

d(t)=C+μlogR(t)−αg(t) (1)

Where *C* is the baseline activity of the dopaminergic neurons when log *R* = *g* = 0, *μ* is the gain, *R* is perceived expected reward, and *α* is the effectiveness of inhibition by the GABAergic output *g*. Note that both the expected reward *R(t)* and the GABAergic output *g(t)* are dynamical, time-dependent variables. Since our model focuses on the regulation of behavior, rather than on learning or representation, we will assume that the log-transformed expected reward log *R* is an input signal that is provided to the circuit. Additionally, while subtractive inhibition was established for VTA dopaminergic neurons, we assume that similar regulation is shared among all midbrain dopaminergic neurons.

To complete the model requires a minimal description of the dynamics of GABAergic output *g*. The mechanisms of interaction between GABAergic and dopaminergic neurons are complex and there are many local and remote interactions [47,48]. However, there are experimental observations that impose constraints on these interactions. Upon presentation of a reward-predicting cue—equivalent to a step increase in *R(t)*—dopamine *d(t)* rapidly increases and then drops and adapts precisely to its baseline on a sub-second timescale [5,26,49] (Fig 2C), a phenomenon called exact adaptation, while GABAergic activity *g(t)* increases to a new steady-state that tracks *R(t)* [49] (Fig 2C).

Exact adaptation is a well-studied property of biological circuits, which can be implemented by a handful of specific feed-forward and feedback mechanisms [50,51]. Since we do not know the mechanistic implementation of the adaptation property in the dopamine circuit, we make the simple assumption of a negative feedback loop. In this design, inhibitory neuron activity *g* is given by an integral-feedback equation with respect to dopamine release *d*:

$$\dot{g}=\omega (\frac{d}{d_{0}}-1)$$ (2)

Or, more generally, $\dot{g}=F(d)$ where *F*(*d*) increases with *d* and has a single zero at *d* = *d*_0_. This feedback loop generates exact adaptation of *d*: the only steady state solution is *d* = *d*_0_, which is the homeostatic activity level of dopaminergic neurons. This is about ~5 spikes/s in mice [30,52]. The parameter *ω* determines the adaptation time of the dopaminergic neurons after a change in *R*(t). This timescale is on the order of hundreds of milliseconds. For the GABAergic neurons, after a step change in *R(t)*, the steady-state firing rate in the model increases proportionally to the logarithm of *R(t)*, such that $g=\frac{C-d_{0}+\mu \log R}{\alpha }$ (this is because GABAergic output integrates dopaminergic activity). Finally, dopamine release represents a temporal-derivative-like computation of *R(t)* as observed by Kim et al. [12] (S1 Fig).

Taken together, Eqs 1 and 2 provide a minimal model for dopamine responses to expected reward inputs *R(t)*. The model is similar to the classic TD-RPE model of dopamine function and can explain the classic observations of prediction error signals that occur during learning. However, there is an important difference: in the TD-RPE model dopamine is activated by expected reward, whereas in our model it is activated by the logarithm of expected reward. For learning *R*, this difference requires a slight modification of the recursive learning rules (Methods). In the following sections we will show that logarithmic sensing has crucial implications for the dynamics of learning and behavioral regulation.

### Model predicts scale-invariant dopamine responses

We now show that the model given by Eqs 1 and 2 can explain one of the most puzzling observations on dopaminergic responses–the scale invariant dopamine responses observed by Tobler et al. [26] (Fig 2D and 2E). In their experiment, Tobler et al. recorded midbrain dopamine neurons of primates presented with a visual cue that predicted liquid rewards with 50% probability. Three cues were presented in pseudorandom order, and each of the cues predicted a reward of a different magnitude over a 10-fold range (0.05ml, 0.15ml, 0.5ml, note that this is in contrast to the case presented in Fig 2B where the same cue was used for all reward magnitudes). Both predictive stimuli and reward reception elicited a positive dopaminergic response, as expected from the TD-RPE theory. However, while the response to the predictive stimuli increased with expected reward magnitude (S2 Fig), the response to the reward itself was invariant despite large differences in reward magnitude. This scale invariance is not consistent with the classical TD-RPE model which predicts that responses to rewards should also increase with reward magnitude [53]. In order to explain this puzzling observation, it has been suggested that there is a normalization process that scales dopamine responses, e.g. according to the standard deviation of the reward distributions [26,53].

Here we show that the observations of Tobler et al. [26] can be explained by our model without invoking any additional normalization process (Fig 2F). The reason for this is that the model has a circuit feature known in systems biology as *fold-change detection (FCD)* [54,55]. FCD is a property where after adaptation the circuit output depends only on relative changes in the input, rather than absolute changes. FCD circuits output the temporal (logarithmic) derivative of low-frequency input signals [56–58]. We therefore call the dopamine model presented here the *dopamine-FCD model* (see *Methods* for a proof that the model has the FCD property).

To see why the FCD property can explain the observations of Tobler et al., [26] consider the input function for each of the cue-reward sequences. When a reward-predicting stimulus appears, the expected reward changes from its previous baseline value in a step-like manner to some value *0*.*5R* that depends on predicted reward magnitude, causing a dopamine response that increases with *R*. At the time point when the reward itself is received, the input function increases by ~2-fold (from *0*.*5R* to *R*, which again for simplicity is modeled as a step increase). Since the dopaminergic response depends only on the fold-change in input, the model predicts identical responses, as observed by Tobler et al. [26]

The FCD property causes the learning and behavior-regulating functions of dopamine to be invariant (i.e., not affected by) multiplying the input field by a scalar [54]–in other words, by multiplying all expected rewards by a constant factor *λ*. The model thus predicts scale-invariance of the dopamine system. This property may be crucial for the dopamine circuit, since rewards can vary widely in magnitude.

### A reward-taxis model for dopamine regulation of behavior

In the following section we will consider whether we can use Eqs 1 and 2 to gain insight into the regulation of behavior by dopamine. To link the dopamine circuit to animal behavior, we first provide an additional equation as a minimal model for dopamine control of motion. The equation is motivated by the well-established role of dopamine as a regulator of action vigor and locomotor activity [13,17,59–61]. The equation posits that dopamine *d* increases movement speed *v*:

$$v=v_{0}\frac{d}{d_{0}}$$ (3)

Where *v*_0_ is movement speed at the homeostatic level *d* = *d*_0_. Dopamine *d* in Eq 3 corresponds to the (normalized) dopaminergic activity in the brain, so that loss of dopaminergic neurons (as occurs in Parkinson’s disease) reduces *d* proportionally, which effectively reduces homeostatic movement speed *v*_0_. We assume a proportional relation between movement speed and *d*, which is consistent with the gradual increase in movement vigor with dopaminergic activity (see Fig 1J and 1K in da Silva et al. [17]).

When the animal is moving towards higher expected rewards, that is, the input field *R(x)* increases as the animal moves, *d* rises above its baseline and movement speed increases; conversely, when the animal moves down *R(x)*, movement speed decreases. The change in *d* may be transient if the change in the input is step-like or gradual (linear); veering away from the reward, however, will result in an undershoot in *d*, and such movements will be inhibited. More generally, since *d* tracks the temporal logarithmic derivative of the input, then as the animal moves its speed will be continuously modulated according the spatial gradients of log *R*(*x*), with movements up the gradient invigorated and movements down the gradient inhibited. This movement regulation, defined by Eq 1–3, results in spending longer times near the peaks of the reward field *R(x)*. We thus call this model *reward-taxis*.

### Reward-taxis quantitatively provides the matching law of operant behavior

**Fig 3:**
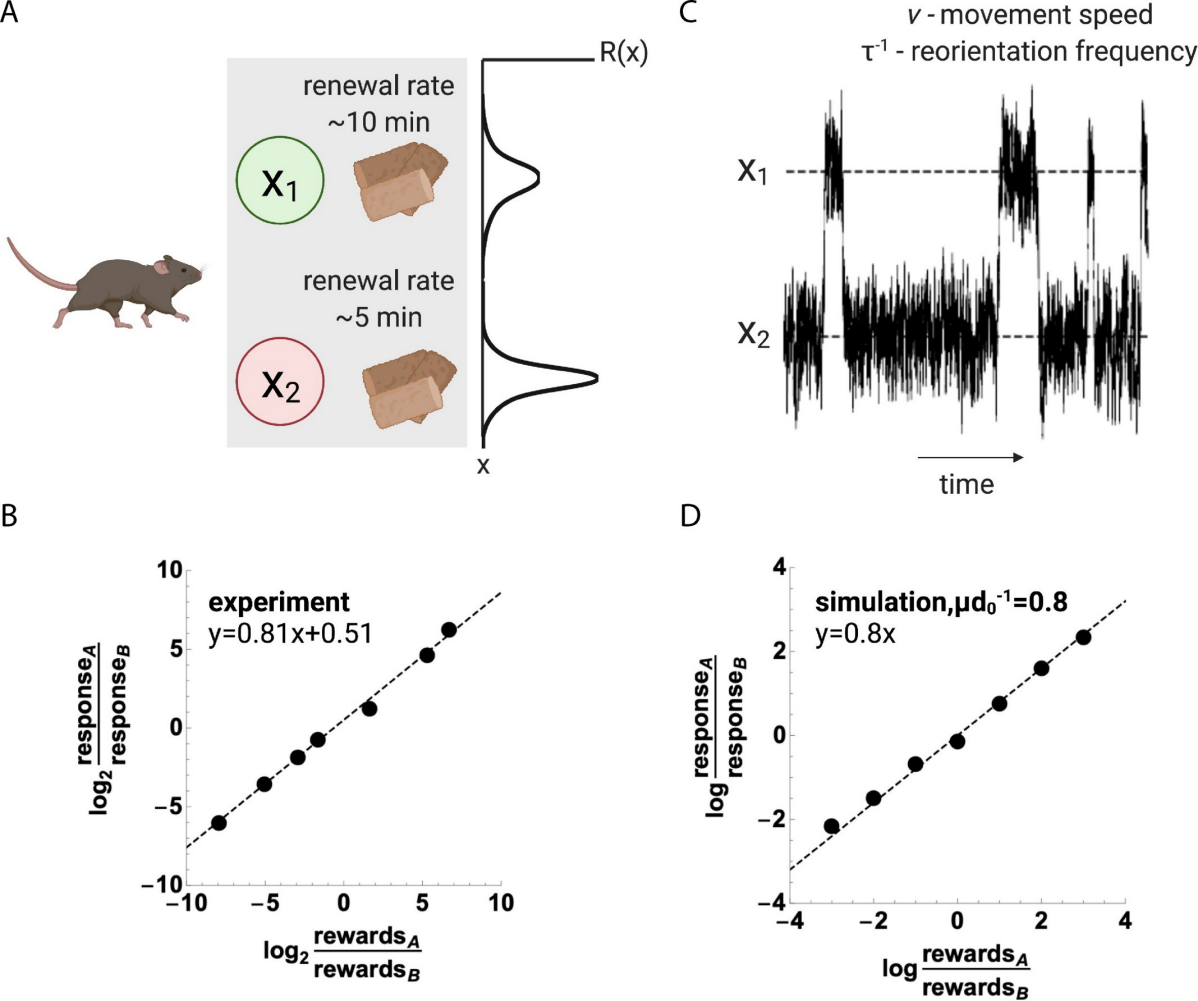
Reward-taxis model provides the generalized matching law. (A) Matching is observed in experiments where the animal can harvest rewards from two locations, which may be baited or empty. Following the consumption of the reward, the location becomes re-baited after some random amount of time, which may be different between the locations. The generalized matching law, amply supported experimentally, is a power-law relation between the reward harvested and the frequency of responses $\frac{P(x_{1})}{P(x_{2})}={\left(\frac{R_{1}}{R_{2}}\right)}^{\beta }$, *R*_1_, *R*_2_ are rewards harvested at locations *A* = *x*_1_, *B* = *x*_2_. Perfect matching occurs for *β* = 1, while undermatching/overmatching are due to variations in *β*. Undermatching, with *β*<1, is often observed, as in (B) (data from Fig 1 of [65]). (C,D) To model stochastic choice behavior of a freely behaving animal, we use a random walk model where the animal moves at speed *v* and reorients at frequency *τ*^−1^, with *v* modulated by dopamine. Random walk model simulation for choice between two expected rewards *R*_1_, *R*_2_. Expected reward input field is the sum of two Gaussians: $R(x)=R_{1}e^{{-\frac{1}{2}(\frac{x-x_{1}}{2b})}^{2}}+R_{2}e^{{-\frac{1}{2}(\frac{x-x_{2}}{b})}^{2}}$ (the exact distribution is not important for matching) with *τ* = 100*ms*, *μ* = 4, *d*_0_ = 5, *v* = 10*cm s*^−1^, *x*_1_ = +30*cm*, *x*_2_ = −30*cm*, *b* = 10*cm* ($\beta =\mu d_{0}^{-1}=0.8$, Methods). An example of a simulation is presented in panel C. The model was simulated for different ratios of *R*_1_/*R*_2_, and the response ratio was estimated by the ratio of the time spent ±2.5*cm* from *A* = *x*_1_, *B* = *x*_2_. Figures were created with BioRender.com.

In the following section we will argue that the reward-taxis model provides a distinct and quantitative explanation for the *general matching law of operant behavior*, one of the best-established behavioral phenomena [62–66,66–70]. Matching is typically observed in concurrent reward schedules where a freely behaving animal harvests rewards that appear stochastically in two separate locations *x*_1_, *x*_2_. The rewards are depleted after harvesting and renew after a random time-interval drawn from a memoryless distribution. In the simplest setting, the same reward is provided in both locations but the average renewal time differs between the locations. In more general settings other parameters (e.g. amount or quality of reinforcement) can vary [71]. There is also usually a cost to switching between options. The matching law, in its time-allocation form [72], posits that the long-term average of the relative amount of time the animal chooses each reward location *P*(*x*_1_), *P*(*x*_2_) goes as a power *β* of the ratio of rewards harvested from the locations *R*_1_, *R*_2_ (Fig 3A and 3B):

$$\frac{P(x_{1})}{P(x_{2})}=k{\left(\frac{R_{1}}{R_{2}}\right)}^{\beta }$$ (4)

where *R*_1_, *R*_2_ correspond to the expected reward at each location (the product of rate and amount of reinforcement [72]). The parameter *k* is a bias term which corresponds to the tendency of the animal to prefer one reward over another even when reinforcement is equal (*R*_1_ = *R*_2_). The bias term typically varies between experiments. The matching law was originally proposed with perfect matching *β* = 1 [73]. A large number of studies in various vertebrate species under different experimental conditions observed that *β* can be somewhat variable, showing slight undermatching (*β*<1) and overmatching (*β*>1), with the former more commonly observed [63–65,68,69,74,75]. Matching has also been observed in wild animal foraging [76,77]. Matching is a robust property which holds over orders of magnitude of reward ratios (up to ~1:500 in pigeons [65]). The overall robustness of the matching law has led authors to suggest that it reflects intrinsic properties of the vertebrate nervous system [69].

Matching is an emergent property of the free behavior of the animal, but its underlying origins are unclear. The continuous, free behavior of the animal contrasts with the discrete choice trials processes that are typical for reinforcement learning models. Matching is not optimal–the optimal policy would be for the animal to regularly switch between the alternatives [78], whereas behavior in experiments is characterized by a memoryless switching process (that is, fixed switching probabilities) [79]. Previous explanations for the general matching law assumed an underlying choice process, such as competition between the groups of neurons representing each reward location [80–83].

Here we provide a novel and distinct explanation for the matching law based on the reward-taxis mechanism (Eqs 1–3). We show that matching is a robust emergent property of the dopamine system, and, moreover, we provide an estimate for *β* in terms of parameters of the dopamine system that can be directly inferred from neuronal recordings (Fig 3C and 3D).

To test whether the model provides the matching law, we model the dynamics of the location of a behaving animal as a stochastic process. Let *R*(*x*) be the input field, which is the expected reward *R* as a function of location *x*. We assume that *R*(*x*) is fixed at *x*_1_, *x*_2_ by the harvested rewards *R*_1_, *R*_2_. To account for the stochastic behavior of the animal in the matching experiments, we model its movement as a biased random walk process, where the animal moves in straight lines at speed *v* (modulated by dopamine) and reorients at random with some fixed probability *τ*^−1^. Allowing the new direction to be correlated with the previous direction does not affect the conclusions, and a model where *τ* (rather than *v*) is modulated by dopamine leads to the same conclusions.

**Fig 4:**
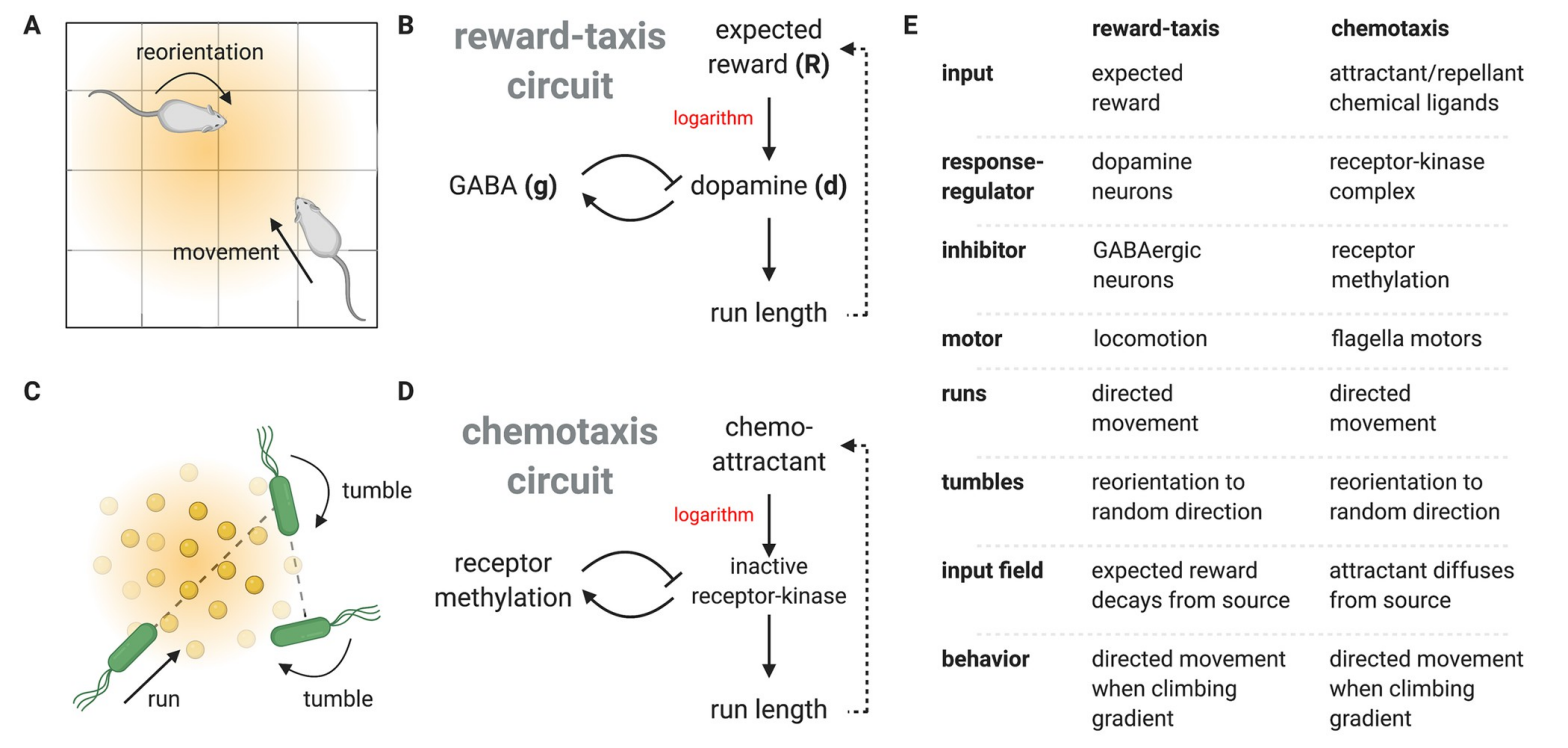
Dopamine regulation of behavior in the model is analogous to bacterial chemotaxis. (A) The behavior of an animal in an open field is modelled as a series of directed movements (runs). The direction of each run is chosen at random (or, more generally, stops between runs decorrelate motion direction), and the duration of each run increases with dopamine level. (B) Dopamine is controlled by an FCD circuit activated by expected reward. (CD) The reward-dopamine-behavior circuit is analogous to the chemotaxis circuit that underlies bacterial navigation towards chemo-attractants. Bacterial motion is composed of a series of runs. The direction of each run is randomized by tumbling events, and run duration increases with the inactivation of a receptor-kinase complex, which is controlled by an FCD circuit activated by chemoattractant concentration. (E) Table detailing the mapping between the dopamine system and the chemotaxis system. Figures were created with BioRender.com.

This biased random walk model is analogous to bacterial chemotaxis: bacteria such as *E*. *coli* use a run-and-tumble navigation strategy to climb gradients of chemical attractants (Fig 4A–4E) [54,56,84,85]. The bacterial chemotaxis signaling network is based on an FCD circuit that controls run duration [54,86]. It therefore maps onto dopamine release dynamics, where expected-reward inputs play the role of chemoattractant concentration in chemotaxis. The key difference is that in chemotaxis the input field results from the diffusion of attractant molecules, whereas in the dopamine system the input field (expected reward) is learned by the animal.

At long time- and length-scales, run-and-tumble motion resembles Brownian motion [87], with a diffusion coefficient *D*≈*m*^−1^*v*^2^*τ*, where *m* is the dimension (we assume that *d* is close to the adapted level *d*_0_). Brownian motion in the model is biased by longer runs when the agent is moving up the gradient. To account for this, one can add to the diffusion process an advection term that is proportional to the logarithmic gradient: χ∇ log *R*(*x*) [88–90]. The advection term corresponds to the average flow at location *x*. Taken together, the stochastic dynamics of the agent are approximated by a Langevin equation similar to the classic Keller-Segel equation used to model chemotaxis [91]:

$$dx=\chi \nabla \log R(x)dt+\sqrt{2D}\;dW$$ (5)

where *W* is an *m*-dimensional Wiener process (see S1 Text for the derivation of Eq 5). For relatively short runs compared with the adaptation time (sub-second timescale), the advection parameter χ, also called chemotactic drift, is given by: $\chi \approx m^{-1}v^{2}\tau \cdot \mu d_{0}^{-1}$ [88], and thus rises with velocity v, gain *μ* and mean run duration *τ*. It is important to note that Eq 5 holds also for the model variant in which dopamine modulates *τ* (see S1 Text).

Eq **5** captures how animal movement depends on the parameters of the dopamine circuit, as well as on movement parameters *τ*, *v*. Decreasing the average run duration *τ* or average movement speed *v* (as in Parkinson’s disease) decreases both diffusivity *D* and advection χ, resulting in slower effective motion and gradient climbing. Gradient climbing efficiency (chemotactic drift) increases with $\mu d_{0}^{-1}$, which is the gain of dopamine neurons normalized by their baseline activity. Other circuit parameters do not affect movement dynamics.

Eq **5** is equivalent to the Langevin Monte Carlo algorithm, a widely-used algorithm from statistical physics for sampling probability distributions and for global optimization [92–95]. The steady-state distribution can be readily solved, using standard methods of statistical physics, similar to a Boltzmann distribution in a potential field. The motion samples a probability distribution *P(x)* proportional to a power *β* of the expected-reward distribution:

$$P(x)\propto e^{\beta \log R(x)}=R{\left(x\right)}^{\beta }$$ (6)

where the power-law *β* equals the normalized gain of the dopaminergic neurons: $\beta =\frac{\chi }{D}=\mu d_{0}^{-1}$. From Eq 6 we infer that for any two expected rewards *R*_1_, *R*_2_ the response rates *P*(*x*_1_), *P*(*x*_2_) obey the general matching law of Eq 4. We note that this results in matching with *k* = 1, but any biases in reward preference, which are multiplicative in *R*_1_ or *R*_2_, will result in a fixed bias term *k*≠1.

The reward-taxis model therefore predicts operant matching with a power law of $\beta =\mu d_{0}^{-1}$. Thus, *β* is equal to the average ratio of gain to baseline activity in dopaminergic neurons. As mentioned above, these values can be estimated from the neuronal measurements of Eshel et al. [30], *μ*≈5 spikes/s and *d*_0_≈5 spikes/s. These values yield *β*≈1, in agreement with the matching law. Similar parameters are found also in primates (S2 Fig). The agreement is striking since there is no a-priori reason for the gain and baseline to be similar; normalized gain $\mu d_{0}^{-1}$ could in principle have a wide range of values including $\mu d_{0}^{-1}\ll 1$ or $\mu d_{0}^{-1}\gg 1$.

The matching exponent *β* only depends on parameters that are intrinsic to the dopaminergic neurons; *β* is independent of movement speed *v* or run duration *τ*, which may vary depending on animal physiology and the environmental context, as well as on the number of dopaminergic neurons. This may explain the robustness of the matching phenomena across species and experimental conditions. The logarithmic derivative property is crucial for obtaining the matching law. A non-logarithmic derivative, or absolute responses, do not provide matching (S1 Text). Taken together, the reward-taxis model can provide a physiological mechanism underpinning operant matching.

Variation in intrinsic neuronal parameters can affect gain *μ* and baseline firing rate *d*_0_. The model predicts that manipulating the relative gain of dopamine neurons $\mu d_{0}^{-1}$ will change the reward sensitivity parameter *β* in the matching law. This prediction can be tested by measuring $\mu d_{0}^{-1}$ in genetically modified animals where matching behavior is different from wild-type. One such case is mice that are deficient in the cannabinoid type-1 receptor (CB^-/-^), which have *β* that is lower by ~30% compared with wild-type mice [96]. In agreement with the model prediction, CB^-/-^ mice also have deficient dopaminergic responses relative to baseline [97].

Other disorders can be due to reduction in the number of functional dopaminergic neurons, as in Parkinson’s disease, where SNc dopaminergic neurons are lost. Such damage is predicted to change *v*_0_. If the damage does not sizably affect the intrinsic properties of surviving neurons, such as gain and baseline, they are not expected to change *β* in matching experiments.

## Discussion

In this study we showed that concepts from systems biology, including exact adaptation, fold-change detection and stochastic navigation, can be mapped to the dopamine system in the brain. We showed that the dopamine circuit may implement a ‘reward-taxis’ mechanism that shares core analogies with bacterial chemotaxis. To show this we developed a mechanistic model of dopamine dynamics based on experimental measurements. The model has similar behavior to the classic TD-RPE model, with a key difference–the circuit is activated by the logarithm of expected reward. The model predicts that dopamine output is invariant to the scale of the distribution of rewards, as observed by Tobler et al. [26], and explains matching in free-operant behavior. Reward-taxis results from the interaction between sensing and movement and implements a simple strategy for climbing gradients of expected reward.

Scale invariance is a recurring motif in biological sensory systems [55]. The model of dopamine transmission as fold-change detection (FCD) of expected reward is thus in line with the conceptualization of dopamine neurons as sensory neurons for reward [98]. FCD includes the classic Weber’s law of sensory systems, which posits that the maximal response to a change in input is normalized by the background level of the signal [99]. FCD is more general than Weber’s law in that the entire dynamics of the output, including amplitude and duration, is normalized by the background input level. FCD allows the system to function in a scale-invariant manner across several decades of background input [54]. It also provides a common scale to compare different types of sensory inputs, by referring to their relative (rather than absolute) changes [100].

While the model focused on the average activity of dopaminergic neurons, the proposed mechanism for FCD (inhibition from neighboring GABAergic neurons) may apply at the level of individual dopaminergic neurons or groups of neurons. This raises the possibility that different dopaminergic neurons could become adapted to different expected-reward levels at the same time, consistent with a recent study that demonstrated that a single reward can simultaneously elicit positive and negative prediction errors in different neurons [101].

The FCD model proposes that RPEs become normalized by the scale of the rewards, but does not account for possible effects of the reward distribution. Such distribution-based effects are evident in the dopamine system. In a recent study, Rothenhoefer et al. showed that rare rewards appear to amplify RPEs more than commonly observed rewards [102]. To further disentangle effects of reward distribution vs. reward scale, we propose the following set of experiments. The FCD model predicts that for a reward schedule with a fixed distribution and a shifted mean *X*, the dopaminergic responses should decay as *X* is increased. To test this we may consider a reward schedule where the animal is randomly rewarded with rewards of magnitudes *r*_1_ = *X*+*Y*, *r*_2_ = *X*−*Y*, preceded by a cue that predicts that some reward will be delivered. The FCD model predicts that dopaminergic responses should decay as *X* increases. For example, when the reward schedule alternates between rewards of magnitude *r*_1_ = 1.5, *r*_2_ = 0.5 (*X* = 1, *Y* = 0.5), the dopaminergic response to the reward of magnitude 1.5 should be larger than the dopaminergic response to a reward of magnitude 11.5 under an alternating schedule with rewards of magnitude *r*_1_ = 11.5, *r*_2_ = 10.5 (*X* = 11, *Y* = 0.5). This contrasts with models based on std. normalization which predict identical responses in both scenarios. Similarly, the FCD model predicts that dopaminergic responses should increase with *Y*, while models based on std. normalization predict identical dopaminergic responses in this scenario as well.

The present study unifies two main effects of dopamine, encoding reward-derivative and increasing movement vigor, by mapping them to a reward-taxis navigational circuit. The circuit is analogous to the bacterial chemotaxis circuit, where in the dopamine case navigation is along gradients of expected reward. The mapping is based on mathematical analogies at both the physiological and behavioral levels. At the physiological level both circuits have the FCD property. At the behavioral level, dopamine increases the probability and vigor of movements, thus increasing the duration of correlated motion (“runs”) compared with reorientations (“tumbles”). Both aspects map to the well-characterized chemotaxis navigation circuit in bacteria.

The stochastic model is sufficient for explaining matching behavior, and provides an accurate mechanistic estimate for the matching constant *β*. The estimate is derived under mild mechanistic assumptions–that movement speed (or run length) is controlled proportionally by dopamine levels, and that run times are relatively short compared with adaptation time. Improved experimental characterization of movement control by dopamine will allow us to relax these assumptions to obtain better estimates for *β*. For example, a nonlinear gain for movement speed regulation by dopamine *v*∝*d*^*h*^ would result in multiplication by a constant prefactor *β* = *hμ*/*d*_0_, and longer run times would result in a proportional decrease in *β* [103]. As long as these effects are mild, we expect our estimate *β*≈1 to hold. Importantly, we still expect the matching constant *β* to be proportional to *μ* and inversely proportional to *d*_0_.

Our study connects between vertebrate motion regulation and the wider family of run-and-tumble stochastic navigation circuits, which includes motion regulation in bacteria, algae, and simple animals [104–107]. Reward-taxis was anticipated in the early work on TD learning, where Montague et al. showed that run-and-tumble dynamics driven by reward prediction errors can explain observations on bee foraging [2].

There are also differences between bacterial chemotaxis and the reward-taxis model for dopamine. The value of *β* in bacterial chemotaxis is much larger than in the dopamine reward-taxis model, with *β*>10 in *E*. *coli* [108,109] and *β*~1 estimated for the dopamine system. The high *β* value in bacteria indicates a strong preference for higher rewards, akin to an optimization for accumulation near attractant peaks. It also allows for collective migration [91]. A value of *β*~1 (which results in the matching law) allows a greater range for exploration of submaximal rewards.

The reward-taxis model was presented for whole-body spatial movement, but its assumptions are general and may potentially extend to other aspects of behavior. One such aspect is *hippocampal replay*, the activation of hippocampal place cells during sharp-wave ripples [110–112]. Hippocampal replay consists of a firing sequence of neurons that represents temporally compressed motion trajectories [111,113,114]. It can occur either during sleep or rest (“offline”) or when the animal is awake and engaged in a task (“online”). Online replay plays an important role in planning and navigation [114]. The activation of the place neurons corresponds to stochastic movement trajectories with a characteristic speed [115] that are biased towards the location of rewards [114]; when foraging is random, the trajectories are diffusive, resembling Brownian motion [112]. Hippocampal activity during online replay is tightly coordinated with reward-associated dopamine transmission [110]. To map reward taxis to hippocampal replay requires that dopamine transmission modulate the stochastic trajectories of hippocampal replay, for example through the modulation of velocity or reorientation frequency.

Another potentially relevant system is eye movements. Eye movements are modulated by dopamine and impaired in Parkinson’s disease [34,116,117], and their vigor is modulated by reward prediction errors [118]. Additionally, random walk models capture gaze dynamics during tasks such as visual search [119–121]. Since eye movements are commonly studied in behavioral experiments such as reward matching, they may be a good candidate to test the reward-taxis model.

While taxis navigation systems in organisms such as E. coli are based on gradients that are created due to diffusion, for the dopamine system the input field is generated by learning—TD learning is sufficient for generating gradients of expected reward. From the point of view of signal processing, TD learning smooths away the high-frequency (or “phasic”) input components [122], leaving a low-frequency input signal that is used for navigation. In this way the dopamine system can allow for gradient-based navigation over fields that are derived from arbitrary sensory inputs.

The *reward-taxis* model does not assume any explicit choice process–in the model, navigation towards regions of higher expected reward is only due to the modulation of movement statistics by dopamine. This may at first sight appear more primitive than standard reinforcement strategies, where the agent compares the expected reward of different alternatives before acting. However, reward-taxis may be advantageous in certain settings. The first advantage is that reward-taxis is computationally cheap–it only requires activation by a single local scalar–which allows for efficient continuous modulation of movements, rather than discrete movement adjustments. The second advantage is that it provides effective sampling of the rewards distributed in the environment by implementing a search algorithm (Eq 5) mathematically analogous to the Langevin Monte Carlo (LMC) algorithm for sampling probability distributions [92–95] and for global optimization [123–128]. Sampling allows the animal to incorporate uncertainty on reward magnitude, probability, or location into its navigation. It also allows the animal to efficiently navigate in complex input fields that include many local minima and maxima [109]. Finally, run-and-tumble navigation provides benefits beyond the Langevin Monte Carlo algorithm by boosting gradient climbing only on sufficiently steep reward gradients due to the positive feedback between behavior and sensing. The positive feedback occurs since running along the gradient provides an increasing input that further enhances the run duration [129]. These advantages suggest that reward-taxis may be a useful strategy when the expected reward input field is complex or uncertain.

The relation between the dopaminergic system and sampling, and in particular the relation between dopaminergic parameters and the matching law parameter *β*, may be relevant to recent findings on dopamine and exploration [130,131]. In mice and humans, dopaminergic antagonists appear to specifically increase random exploration, rather than affect learning [130,131]. Under our modelling framework, this effect may correspond to a decrease in *β* by the treatment, for example, due to a reduction in the effective dopaminergic gain. This would result in altered behavioral output, without necessarily affecting learning. More generally, the sampling framework can provide a quantitative theoretical framework to model the relation between dopamine and various aspects of stochastic exploration, such as novelty-driven exploration [132,133].

A more realistic and complete model would include other aspects of decision making such as goal-directed behavior and planning. It is important to note that since the FCD model does not hinder learning, it is compatible with these aspects and they are likely to complement reward-taxis with more directed movement. Such a combination of navigation mechanisms is evident also in simple organisms that employ run-and-tumble navigation. For example, in *C*. *elegans* thermotaxis, run-and-tumble navigation is combined with biased reorientations in order to navigate towards an optimal temperature range [106]. Formally, while run-and-tumble navigation resembles Langevin-based sampling, directed reorientations are more closely related to gradient descent, which is efficient for local optimization but poor for global optimization [127,134,135]. We thus propose that the reward-taxis mechanism we describe can complement other navigation and decision making-mechanisms to allow for efficient navigation in complex environments.

## Methods

### Model equations and fold-change detection

The equations for dopamine (d) and GABAergic inhibition (g) are provided by:

$$\dot{d}=\omega_{d}(C+\mu \log R-\alpha g-d)$$ (7)

$$\dot{g}=\omega (\frac{d}{d_{0}}-1)$$ (8)

For the dopamine equation, *ω*_*d*_ determines the dopamine degradation rate, *μ* is dopamine gain, *R* is expected reward (defined in the next Methods section), and *α* is GABAergic inhibition strength. For the GABAergic inhibition equation, *ω* determines the adaptation rate and *d*_0_ is the adapted steady-state of dopamine. For simplicity, we assume that dopamine dynamics are faster than the dynamics of adaptation due to *g* (i.e., *ω*_*d*_ is large compared with *ω*, this assumption is not important for our conclusions) so we take:

d=C+μlogR−αg (9)

Eqs 7, 8 and 9 can represent the average activity of individual neurons, or the total activity of many neurons. We therefore used the same equations both to model average individual neuron recordings (as in Fig 2), and to model the effect of dopamine on movement, which is likely to be the sum of the activity of many neurons.

Consider now a constant input *R* = *R*_0_, so that after some time the system reaches steady-state. To find the steady state, we solve Eqs 7 and 8, taking $\dot{d}=0,\dot{g}=0$, which yields the steady-state solutions *d*_*st*_ = *d*_0_ and *g*_*st*_ = *α*^−1^(*C*−*d*_0_+*μ* log *R*_0_). The observation that *d*_*st*_ = *d*_0_ regardless of *R*_0_ and other circuit parameters is an important circuit feature from systems biology known as *exact adaptation* [27,50,136–138]. This feature is essential for explaining why dopamine activity returns precisely to baseline after a step increase in expected reward, while GABAergic activity increases in a way that tracks expected reward.

Beyond exact adaptation, the system has an even stronger property of *fold-change detection* (FCD). FCD is defined as dynamics of dopamine (*d*) in response to an input *λR*(*t*) that are independent of *λ*, starting from initial conditions at steady-state for *λR*(0). To show this we relabel *g* = *g*′+*α*^−1^ *μ* log *λ*:

$$\dot{d}=\omega_{d}(C+\mu \log\lambda R-\alpha g-d)=\omega_{d}(C+\mu \log R+\mu \log\lambda -\alpha g-d)=\omega_{d}(C+\mu \log R-\alpha g^{\prime }-d)$$ (10)

$$\dot{g^{\prime }}=\omega (\frac{d}{d_{0}}-1)$$ (11)

Note that the rate equation for $\dot{g^{\prime }}$ is the same as for $\dot{g}$ since $\frac{dg^{\prime }}{dt}=\frac{dg\prime }{dg}\frac{dg}{dt}=\frac{dg}{dt}$. Eqs 10 and 11 are completely independent of *λ*, and their steady-state $g_{st}^{\prime }=\alpha^{-1}(C-d_{0}+\mu \log R_{0})$ and *d*_*st*_ = *d*_0_ is also independent of *λ*. This means that the dynamics of the system have the FCD property. The FCD property is essential for explaining the scale invariance of the dopaminergic responses to rewards in Fig 2 –the response only depends on the fold-change of expected reward (two-fold change upon reception of reward at p = 0.5) but not on reward magnitude.

While Eqs 7, 8 and 9 provide FCD, they are not the only possible model that provides FCD for this system. In particular, a feed-forward model where expected reward activates *g* is also possible, i.e.:

$$\dot{d}=\omega_{d}(d_{0}+C+\mu \log R-\alpha g-d)$$ (12)

$$\dot{g}=\omega (\frac{C+\mu \log R}{\alpha }-g)$$ (13)

For this circuit, the steady state for a constant input *R* = *R*_0_ is $g_{st}=\frac{a+\mu \log R_{0}}{\alpha }$ and *d*_*st*_ = *d*_0_. FCD can also be analogously shown. Given an input *λr*(*t*), we can take *g* = *g*′+*α*^−1^ *μ* log *λ*, which again provides equations and steady-state that are independent of *λ*:

$$\dot{d}=\omega_{d}(d_{0}+C+\mu \log\lambda R-\alpha g-d)=\omega_{d}(d_{0}+C+\mu \log R-\alpha g^{\prime }-d)$$ (14)

$$\dot{g^{\prime }}=\omega (\frac{C+\mu \log\lambda R}{\alpha }-g)=\omega (\frac{C+\mu \log R}{\alpha }-g\prime )$$ (15)

While this simple log-linear model captures various important experimental observations, it is important to note that it has some clear limitations. One limitation is that both *d* and *g* can in principle reach negative values when *R* is small. Measurements of dopamine responses in monkeys indeed show deviations from sub-linearity for small rewards [139]. The model can be adjusted to prevent negative undershoots (S3 Fig). Future studies may build on improved measurements and better mechanistic characterization of the dopamine circuit to refine this model. Finally, the original studies quantifying the input/output relation between reward magnitude and dopaminergic output, presented in Fig 2, considered fits by strongly sublinear power- and hill-functions [30,38]. It is not possible to discriminate between these functions and the logarithmic relation with the available data, and such a fit would require more accurate measurements over large magnitude ranges.

### Definition of expected reward and relation between the circuit and TD learning

Here we will define the input to the circuit, which is the logarithmic expected reward log *R*, and present it in the context of the temporal difference (TD) learning theory of dopamine function [5,7]. We first define the expected temporally discounted sum of future rewards *V* (also known as the value function in TD learning):

$$V(t_{0})=E[\sum_{t=t_{0}}^{\infty }\gamma^{t}r(t)dt]$$ (16)

Where *γ*<1 is a “future discounting” factor and *r*(*t*) is the reward received at time *t* into the future (here for simplicity we take discrete time; for equivalent formulation for continuous time, see Doya [140]). It is possible to think of *V* as a function of the current state *s* of the agent, which may include for example its location in space *x*. This is known as the Markovian setting, where we denote the value function as *V*(*s*). The value function plays an important role in decision making—learning the value function is a principal focus of reinforcement learning algorithms [5,7].

In our model, the input to the circuit for an agent moving into a state *s* at time *t* is defined using the expected reward *R*:

R(t,s)=r(t)+V(s)=r(t)+V(t) (17)

As an example, consider the setting of Fig 2D–2F, where a reward of size *r* = *y* is delivered with probability *p* at Δt time-units into future: the expected reward would in this case be *R*(0)≈*pγ*^Δt^*y*. An actual delivery of the reward would then increase *R* to *R*(Δ*t*)≈*y*, so the ratio $\frac{R(\Delta t)}{R(0)}=\frac{1}{p\gamma^{\Delta t}}$ is independent of reward magnitude.

Note that due to discounting and uncertainty, *R* decays with the distance from a location where a reward is delivered, as in Fig 1D [36].

We will now show that our model is consistent with the TD learning theory of dopamine function with a slight modification to the TD learning rule. We will first briefly present the TD learning algorithm. In reinforcement learning, the agent usually does not know *V* and needs to learn it from experience. This presents a computational problem, since *V* is an infinite sum over unknown future events. A way to get around this is to update the learned *V* using dynamic programming [141]. The key insight is that Eq 16 can be rewritten as:

$$V(t_{0})=E[\sum_{t=t_{0}}^{\infty }\gamma^{t}r(t)dt]=E[r(t_{0})]+\gamma V(t_{0}+1)$$ (18)

The above equation implies that *V* can be estimated iteratively with a simple update rule, which is at the heart of TD learning. If the agent is at state *s* at time *t*, and at state *s’* at time *t+1*, the update rule is:

$$V(t+1,s)\leftarrow V(t,s)+\alpha \underset{prediction\;error}{\underset{︸}{\left(r(t)+\gamma V(t,s^{\prime })-V(t+1,s)\right)}}$$ (19)

Where *V*(*t*, *s*) is the computed estimate of the expected reward at state *s* at time *t*, *α* is the learning rate, *r*(t) is the reward delivered at time *t* and *γ* is the discounting factor. There is extensive literature demonstrating correspondence between TD learning and midbrain dopamine function (reviewed by [8]); specifically, experiments show a correspondence between phasic dopamine secretion and the prediction error term of Eq 19 [5,8], in the sense that positive or negative firing of dopamine neurons relative to baseline corresponds positive and negative predictions errors in TD models of learning.

We will now show that our model is capable of learning the logarithm of *V* (that is, the logarithm of the entire discounted sum over future rewards), in a manner similar to the learning of *V* by classic TD learning. Since both are equivalent, our model is sufficient for explaining TD learning by dopaminergic responses. For this we will develop a plausible temporal difference learning rule based on logarithmic prediction errors: $\delta_{\log}=\log(r(t)+\gamma V(t,s^{\prime }))-\log V(t,s)$. The learning rule is an extension of the learning rule presented in Eqs 15–17 in Coulthard et al. [142]. To devise the learning rule, consider the Taylor expansion of the logarithm of the update rule given in Eq 19 around *r*(*t*)+*γV*(*t*, *s*′) = *V*(*t*, *s*):

$$\begin{array}{l} \log V(t+1,s)\leftarrow \log(V(t,s)+\alpha (r(t)+\gamma V(t,s^{\prime })-V(t,s)))\approx \log V(t,s)+\\ \alpha (\frac{r(t)+\gamma V(t,s^{\prime })}{V(t,s)}-1)=\log V(t,s)+\alpha (e^{\log(r(t)+\gamma V(t,s^{\prime }))-\log V(t,s)}-1)=\log V(t,s)+\\ \alpha (e^{\delta_{\log}}-1) \end{array}$$ (20)

The above equation represents an update rule that only needs the modified prediction error term *δ*_log_ in order to learn the value function. In the continuous limit, and in the absence of reward, the error term is approximately proportional the logarithmic derivative of the value function. This corresponds to the output of our proposed FCD model for low frequency signals.

The output of the circuit to a delivered reward in a transition from a state s to a state s’ is also approximately proportional to the above error term:

$$\Delta d=C+\mu \log R(t)-\alpha g(t)-d_{0}=\mu (\log\left(r(t)+V(s^{'})\right)-\log\left(V(s\right))$$ (21)

The final equality is due to the fact that prior to reward delivery, GABAergic output adapts to *α*^−1^(*C*−*d*_0_+*μ* log *V*(*s*)). The FCD model is therefore compatible with the TD learning theory of dopamine function. In S4 Fig we provide simulations for learning with the modified learning rule, where we show that it indeed learns log *V*.

### Analysis

The fit of the dopaminergic responses in Fig 2 (including confidence intervals) was performed using the NonlinearModelFit function of Mathematica (version 12.1.1). All other figures and simulations were produced using Python (version 3.8.5). The source code and data to produce all the figures is available at https://github.com/omerka-weizmann/reward_taxis.

## Supporting information

S1 Text Supplementary theory, including derivation of Langevin dynamics and matching law from run-and-tumble model. (DOCX)

S1 FigModel dynamics are consistent with derivative-like dopamine dynamics on a seconds timescale.Dopamine output to movement in a reward gradient given by $R(x)=e^{-\gamma x^{h}}$, with *h* = 1.5, *x*_0_ = 1, *v*_0_ = 1 and *γ* = 0.04, and perturbations as described in Fig 2 of [12]. *Insets*. Corresponding dopaminergic outputs from mice (left to right: n = 11, n = 11, n = 15, n = 5) VTA neurons measured by calcium imaging, from Fig 2C, 2G, 2K and 2O in [12], smoothed using a Savitzky–Golay filter. All simulations were performed with the parameters provided in Table 1.(DOCX)

S2 Fig Dopaminergic responses to variable size liquid rewards in monkeys. Dopamine responses in Macaque monkeys to cues predicting variable size liquid rewards (dashed lines) correspond to model simulations, given by a step *R*(*t*) = *R*_0_+*λuθ*(*t*−*t*_0_) where *θ*(*t*−*t*_0_) is a unit step function, *R*_0_ = 1 and *λ* = 20 *ml*^−1^, and *u* is the expected value of the liquid volume that the cue predicts. Data is from the population neuron recordings of Fig 1B in Tobler et al. [26], corresponding to, from left to right: 0.0 ml with probability *p = 1*, 0.05 ml with probability *p = 0*.*5*, 0.15 ml with probability *p = 0*.*5*, 0.15 ml with probability *p = 1*, and 0.5 ml with probability *p = 0*.*5*. The probabilistic responses correspond to the responses where scale invariance is observed in Fig 2.
(DOCX)

S3 FigResponses to reward gain / omission in unadjusted and adjusted FCD models.(A) Reward reception and omission was simulated according to Eqs 1,2 in the manuscript in a manner similar to the simulations in Fig 1. The input is given by *R*(*t*) = *R*_0_+*λθ*(*t*−*t*_0_) where *θ*(*t*−*t*_0_) is a unit step function, *R*_0_ = 1 and *λ* = 7 for the reward reception and *R*_0_ = 7, *λ* = −6 for reward omission. Note that Eq 1 reaches negative values upon reward omission. (B) Dynamics for model where Eq 1 is adjusted as $d=C+\mu \;\log R-\alpha g\frac{d}{d+k_{d}}$ (here taking *k*_*d*_ = 1). This model does not reach negative values of *d*, and behaves similarly to the FCD model if *k*_*d*_≪*d*_0_.(DOCX)

S4 FigLearning the logarithm of expected rewards with recursive rules.(A) Simulation setup. The agent progresses through a series of *N* states S1,…,SN, where in the final stage SN a reward is drawn according to a distribution with a fixed mean reward value. In the simulations we use three distributions (deterministic reward, normal distribution with CV = 0.3, and Bernoulli trials). A logarithmic value function log *V* is learned recursively according to the rule $\log V_{t+1}(s)\leftarrow \log V_{t}(s)+\alpha (e^{\log\left(r(t)+\gamma V_{t}(s+1)\right)-\log V_{t}(s)}-1)$. (B,C) Learning simulations with reward magnitude 50 (B) and 200 (C). Thick line denotes a log *V*_*t*+1_(*S*1) in a single simulation, while thin dashed line denotes expected $\log V_{t+1}(s)=\log E[\sum_{t=t_{0}}^{\infty }\gamma^{t}r(t)dt]$. Simulation parameters are *N* = 5, *α* = 0.02. Figures were created with BioRender.com.(DOCX)

## References

[1] Barto AG. Adaptive critics and the basal ganglia. 1995.

[2] Montague PR, Dayan P, Person C, Sejnowski TJ. Bee foraging in uncertain environments using predictive hebbian learning. Nature. 1995;377: 725–728. doi: 10.1038/377725a0 7477260

[3] Houk JC, Davis JL, Beiser DG. Models of information processing in the basal ganglia. MIT press; 1995.

[4] Montague PR, Dayan P, Sejnowski TJ. A framework for mesencephalic dopamine systems based on predictive Hebbian learning. J Neurosci. 1996;16: 1936–1947. doi: 10.1523/JNEUROSCI.16-05-01936.1996 8774460 PMC6578666

[5] Schultz W, Dayan P, Montague PR. A neural substrate of prediction and reward. Science. 1997;275: 1593–1599. doi: 10.1126/science.275.5306.1593 9054347

[6] Steinberg EE, Keiflin R, Boivin JR, Witten IB, Deisseroth K, Janak PH. A Causal Link Between Prediction Errors, Dopamine Neurons and Learning. Nat Neurosci. 2013;16: 966–973. doi: 10.1038/nn.3413 23708143 PMC3705924

[7] Sutton RS, Barto AG. Introduction to reinforcement learning. MIT press Cambridge; 1998.

[8] Glimcher PW. Understanding dopamine and reinforcement learning: the dopamine reward prediction error hypothesis. Proc Natl Acad Sci. 2011;108: 15647–15654. doi: 10.1073/pnas.1014269108 21389268 PMC3176615

[9] Howe MW, Tierney PL, Sandberg SG, Phillips PE, Graybiel AM. Prolonged dopamine signalling in striatum signals proximity and value of distant rewards. nature. 2013;500: 575–579. doi: 10.1038/nature12475 23913271 PMC3927840

[10] Hamid AA, Pettibone JR, Mabrouk OS, Hetrick VL, Schmidt R, Vander Weele CM, et al. Mesolimbic dopamine signals the value of work. Nat Neurosci. 2016;19: 117–126. doi: 10.1038/nn.4173 26595651 PMC4696912

[11] Mohebi A, Pettibone JR, Hamid AA, Wong J-MT, Vinson LT, Patriarchi T, et al. Dissociable dopamine dynamics for learning and motivation. Nature. 2019;570: 65–70. doi: 10.1038/s41586-019-1235-y 31118513 PMC6555489

[12] Kim HR, Malik AN, Mikhael JG, Bech P, Tsutsui-Kimura I, Sun F, et al. A Unified Framework for Dopamine Signals across Timescales. Cell. 2020 [cited 28 Nov 2020]. doi: 10.1016/j.cell.2020.11.013 33248024 PMC7736562

[13] Niv Y, Daw ND, Joel D, Dayan P. Tonic dopamine: opportunity costs and the control of response vigor. Psychopharmacology (Berl). 2007;191: 507–520. doi: 10.1007/s00213-006-0502-4 17031711

[14] Mazzoni P, Hristova A, Krakauer JW. Why don’t we move faster? Parkinson’s disease, movement vigor, and implicit motivation. J Neurosci. 2007;27: 7105–7116. doi: 10.1523/JNEUROSCI.0264-07.2007 17611263 PMC6794577

[15] Berridge KC. The debate over dopamine’s role in reward: the case for incentive salience. Psychopharmacology (Berl). 2007;191: 391–431. doi: 10.1007/s00213-006-0578-x 17072591

[16] Dudman JT, Krakauer JW. The basal ganglia: from motor commands to the control of vigor. Curr Opin Neurobiol. 2016;37: 158–166. doi: 10.1016/j.conb.2016.02.005 27012960

[17] da Silva JA, Tecuapetla F, Paixão V, Costa RM. Dopamine neuron activity before action initiation gates and invigorates future movements. Nature. 2018;554: 244–248. doi: 10.1038/nature25457 29420469

[18] Shadmehr R, Ahmed AA. Vigor: neuroeconomics of movement control. MIT Press; 2020. 10.1017/S0140525X20000667 33261698

[19] Meder D, Herz DM, Rowe JB, Lehéricy S, Siebner HR. The role of dopamine in the brain-lessons learned from Parkinson’s disease. Neuroimage. 2019;190: 79–93. doi: 10.1016/j.neuroimage.2018.11.021 30465864

[20] Berke JD. What does dopamine mean? Nat Neurosci. 2018;21: 787–793. doi: 10.1038/s41593-018-0152-y 29760524 PMC6358212

[21] Friston K. The free-energy principle: a unified brain theory? Nat Rev Neurosci. 2010;11: 127–138. doi: 10.1038/nrn2787 20068583

[22] Bogacz R. Dopamine role in learning and action inference. Elife. 2020;9: e53262. doi: 10.7554/eLife.53262 32633715 PMC7392608

[23] Niv Y, Daw N, Dayan P. How fast to work: Response vigor, motivation and tonic dopamine. Adv Neural Inf Process Syst. 2005;18: 1019–1026.

[24] Yoon T, Geary RB, Ahmed AA, Shadmehr R. Control of movement vigor and decision making during foraging. Proc Natl Acad Sci. 2018;115: E10476–E10485. doi: 10.1073/pnas.1812979115 30322938 PMC6217431

[25] Daw ND, O’doherty JP, Dayan P, Seymour B, Dolan RJ. Cortical substrates for exploratory decisions in humans. Nature. 2006;441: 876–879. doi: 10.1038/nature04766 16778890 PMC2635947

[26] Tobler PN, Fiorillo CD, Schultz W. Adaptive Coding of Reward Value by Dopamine Neurons. Science. 2005;307: 1642–1645. doi: 10.1126/science.1105370 15761155

[27] Alon U. An introduction to systems biology: design principles of biological circuits. CRC press; 2019.

[28] Schultz W. Predictive reward signal of dopamine neurons. J Neurophysiol. 1998;80: 1–27. doi: 10.1152/jn.1998.80.1.1 9658025

[29] Brischoux F, Chakraborty S, Brierley DI, Ungless MA. Phasic excitation of dopamine neurons in ventral VTA by noxious stimuli. Proc Natl Acad Sci. 2009;106: 4894–4899. doi: 10.1073/pnas.0811507106 19261850 PMC2660746

[30] Eshel N, Tian J, Bukwich M, Uchida N. Dopamine neurons share common response function for reward prediction error. Nat Neurosci. 2016;19: 479–486. doi: 10.1038/nn.4239 26854803 PMC4767554

[31] Parker NF, Cameron CM, Taliaferro JP, Lee J, Choi JY, Davidson TJ, et al. Reward and choice encoding in terminals of midbrain dopamine neurons depends on striatal target. Nat Neurosci. 2016;19: 845–854. doi: 10.1038/nn.4287 27110917 PMC4882228

[32] Lee RS, Mattar MG, Parker NF, Witten IB, Daw ND. Reward prediction error does not explain movement selectivity in DMS-projecting dopamine neurons. Behrens TE, Schoenbaum G, Schoenbaum G, Willuhn I, editors. eLife. 2019;8: e42992. doi: 10.7554/eLife.42992 30946008 PMC6464606

[33] Engelhard B, Finkelstein J, Cox J, Fleming W, Jang HJ, Ornelas S, et al. Specialized coding of sensory, motor and cognitive variables in VTA dopamine neurons. Nature. 2019;570: 509–513. doi: 10.1038/s41586-019-1261-9 31142844 PMC7147811

[34] Kori A, Miyashita N, Kato M, Hikosaka O, Usui S, Matsumura M. Eye movements in monkeys with local dopamine depletion in the caudate nucleus. II. Deficits in voluntary saccades. J Neurosci. 1995;15: 928–941. doi: 10.1523/JNEUROSCI.15-01-00928.1995 7823190 PMC6578280

[35] Matsumoto M, Hikosaka O. Two types of dopamine neuron distinctly convey positive and negative motivational signals. Nature. 2009;459: 837–841. doi: 10.1038/nature08028 19448610 PMC2739096

[36] Gershman SJ. Dopamine ramps are a consequence of reward prediction errors. Neural Comput. 2014;26: 467–471. doi: 10.1162/NECO_a_00559 24320851

[37] Daw ND, Tobler PN. Chapter 15—Value Learning through Reinforcement: The Basics of Dopamine and Reinforcement Learning. In: Glimcher PW, Fehr E, editors. Neuroeconomics (Second Edition). San Diego: Academic Press; 2014. pp. 283–298. doi: 10.1016/B978-0-12-416008-8.00015–2

[38] Eshel N, Bukwich M, Rao V, Hemmelder V, Tian J, Uchida N. Arithmetic and local circuitry underlying dopamine prediction errors. Nature. 2015;525: 243–246. doi: 10.1038/nature14855 26322583 PMC4567485

[39] Dehaene S. The neural basis of the Weber–Fechner law: a logarithmic mental number line. Trends Cogn Sci. 2003;7: 145–147. doi: 10.1016/s1364-6613(03)00055-x 12691758

[40] Nieder A, Miller EK. Coding of cognitive magnitude: Compressed scaling of numerical information in the primate prefrontal cortex. Neuron. 2003;37: 149–157. doi: 10.1016/s0896-6273(02)01144-3 12526780

[41] Shen J. On the foundations of vision modeling: I. Weber’s law and Weberized TV restoration. Phys Nonlinear Phenom. 2003;175: 241–251.

[42] Dehaene S, Izard V, Spelke E, Pica P. Log or linear? Distinct intuitions of the number scale in Western and Amazonian indigene cultures. Science. 2008;320: 1217–1220. doi: 10.1126/science.1156540 18511690 PMC2610411

[43] Nieder A, Dehaene S. Representation of number in the brain. Annu Rev Neurosci. 2009;32: 185–208. doi: 10.1146/annurev.neuro.051508.135550 19400715

[44] Laughlin SB. The role of sensory adaptation in the retina. J Exp Biol. 1989;146: 39–62. doi: 10.1242/jeb.146.1.39 2689569

[45] Bernoulli D. Specimen theoriae novae de mensura sortis. Gregg; 1968.

[46] Rubinstein M. The strong case for the generalized logarithmic utility model as the premier model of financial markets. Financial Dec Making Under Uncertainty. Elsevier; 1977. pp. 11–62.

[47] Morales M, Margolis EB. Ventral tegmental area: cellular heterogeneity, connectivity and behaviour. Nat Rev Neurosci. 2017;18: 73–85. doi: 10.1038/nrn.2016.165 28053327

[48] Cox J, Witten IB. Striatal circuits for reward learning and decision-making. Nat Rev Neurosci. 2019;20: 482–494. doi: 10.1038/s41583-019-0189-2 31171839 PMC7231228

[49] Cohen JY, Haesler S, Vong L, Lowell BB, Uchida N. Neuron-type-specific signals for reward and punishment in the ventral tegmental area. nature. 2012;482: 85–88. doi: 10.1038/nature10754 22258508 PMC3271183

[50] Ma W, Trusina A, El-Samad H, Lim WA, Tang C. Defining network topologies that can achieve biochemical adaptation. Cell. 2009;138: 760–773. doi: 10.1016/j.cell.2009.06.013 19703401 PMC3068210

[51] Adler M, Szekely P, Mayo A, Alon U. Optimal regulatory circuit topologies for fold-change detection. Cell Syst. 2017;4: 171–181. doi: 10.1016/j.cels.2016.12.009 28089543

[52] Robinson S, Smith DM, Mizumori SJY, Palmiter RD. Firing properties of dopamine neurons in freely moving dopamine-deficient mice: Effects of dopamine receptor activation and anesthesia. Proc Natl Acad Sci. 2004;101: 13329–13334. doi: 10.1073/pnas.0405084101 15317940 PMC516529

[53] Gershman SJ. Dopamine, inference, and uncertainty. Neural Comput. 2017;29: 3311–3326. doi: 10.1162/neco_a_01023 28957023

[54] Shoval O, Goentoro L, Hart Y, Mayo A, Sontag E, Alon U. Fold-change detection and scalar symmetry of sensory input fields. Proc Natl Acad Sci. 2010;107: 15995–16000. doi: 10.1073/pnas.1002352107 20729472 PMC2936624

[55] Adler M, Alon U. Fold-change detection in biological systems. Curr Opin Syst Biol. 2018;8: 81–89.

[56] Tu Y, Shimizu TS, Berg HC. Modeling the chemotactic response of Escherichia coli to time-varying stimuli. Proc Natl Acad Sci. 2008;105: 14855–14860. doi: 10.1073/pnas.0807569105 18812513 PMC2551628

[57] Adler M, Mayo A, Alon U. Logarithmic and power law input-output relations in sensory systems with fold-change detection. PLoS Comput Biol. 2014;10: e1003781. doi: 10.1371/journal.pcbi.1003781 25121598 PMC4133048

[58] Lang M, Sontag E. Scale-invariant systems realize nonlinear differential operators. 2016 American Control Conference (ACC). IEEE; 2016. pp. 6676–6682.

[59] Beierholm U, Guitart-Masip M, Economides M, Chowdhury R, Düzel E, Dolan R, et al. Dopamine modulates reward-related vigor. Neuropsychopharmacology. 2013;38: 1495–1503. doi: 10.1038/npp.2013.48 23419875 PMC3682144

[60] Panigrahi B, Martin KA, Li Y, Graves AR, Vollmer A, Olson L, et al. Dopamine is required for the neural representation and control of movement vigor. Cell. 2015;162: 1418–1430. doi: 10.1016/j.cell.2015.08.014 26359992

[61] Ek F, Malo M, Åberg Andersson M, Wedding C, Kronborg J, Svensson P, et al. Behavioral Analysis of Dopaminergic Activation in Zebrafish and Rats Reveals Similar Phenotypes. ACS Chem Neurosci. 2016;7: 633–646. doi: 10.1021/acschemneuro.6b00014 26947759

[62] Herrnstein RJ. On the law of effect 1. J Exp Anal Behav. 1970;13: 243–266. doi: 10.1901/jeab.1970.13-243 16811440 PMC1333768

[63] Baum WM. On two types of deviation from the matching law: bias and undermatching 1. J Exp Anal Behav. 1974;22: 231–242. doi: 10.1901/jeab.1974.22-231 16811782 PMC1333261

[64] Baum WM. Optimization and the matching law as accounts of instrumental behavior. J Exp Anal Behav. 1981;36: 387–403. doi: 10.1901/jeab.1981.36-387 16812255 PMC1333108

[65] Baum WM, Schwendiman JW, Bell KE. Choice, contingency discrimination, and foraging theory. J Exp Anal Behav. 1999;71: 355–373. doi: 10.1901/jeab.1999.71-355 16812900 PMC1284717

[66] Sugrue LP, Corrado GS, Newsome WT. Matching behavior and the representation of value in the parietal cortex. science. 2004;304: 1782–1787. doi: 10.1126/science.1094765 15205529

[67] Dallery J, Soto PL. Herrnstein’s hyperbolic matching equation and behavioral pharmacology: Review and critique. Behav Pharmacol. 2004;15: 443–459. doi: 10.1097/00008877-200411000-00001 15472567

[68] Lau B, Glimcher PW. Dynamic response-by-response models of matching behavior in rhesus monkeys. J Exp Anal Behav. 2005;84: 555–579. doi: 10.1901/jeab.2005.110-04 16596980 PMC1389781

[69] McDowell JJ. On the theoretical and empirical status of the matching law and matching theory. Psychol Bull. 2013;139: 1000. doi: 10.1037/a0029924 22946881

[70] Houston AI, Trimmer PC, McNamara JM. Matching Behaviours and Rewards. Trends Cogn Sci. 2021. doi: 10.1016/j.tics.2021.01.011 33612384

[71] Davison M, McCarthy D. The matching law: a research review. Hillsdale, N.J: L. Erlbaum; 1988.

[72] Baum WM, Rachlin HC. Choice as time allocation 1. J Exp Anal Behav. 1969;12: 861–874. doi: 10.1901/jeab.1969.12-861 16811415 PMC1338696

[73] Herrnstein RJ. Relative and absolute strength of response as a function of frequency of reinforcement. J Exp Anal Behav. 1961;4: 267. doi: 10.1901/jeab.1961.4-267 13713775 PMC1404074

[74] William BM. Matching, undermatching, and overmatching in studies of choice. J Exp Anal Behav. 1979;32: 269–281. doi: 10.1901/jeab.1979.32-269 501274 PMC1332902

[75] Davison M. Choice, changeover, and travel: A quantitative model. J Exp Anal Behav. 1991;55: 47–61. doi: 10.1901/jeab.1991.55-47 16812630 PMC1322977

[76] Baum WM. Choice in free-ranging wild pigeons. Science. 1974;185: 78–79. doi: 10.1126/science.185.4145.78 17779288

[77] Houston A. THE MATCHING LAW APPLIES TO WAGTAILS’FORAGING IN THE WILD. J Exp Anal Behav. 1986;45: 15–18. doi: 10.1901/jeab.1986.45-15 16812441 PMC1348207

[78] Houston AI, McNamara J. How to maximize reward rate on two variable-interval paradigms. J Exp Anal Behav. 1981;35: 367–396. doi: 10.1901/jeab.1981.35-367 16812223 PMC1333091

[79] Heyman GM. A MARKOV MODEL DESCRIPTION OF CHANGEOVER PROBABILITIES ON CONCURRENT VARIABLE-INTERVAL SCHEDULES 1. J Exp Anal Behav. 1979;31: 41–51. doi: 10.1901/jeab.1979.31-41 16812122 PMC1332788

[80] Herrnstein RJ, Prelec D. Melioration: A theory of distributed choice. J Econ Perspect. 1991;5: 137–156.

[81] Soltani A, Wang X-J. A biophysically based neural model of matching law behavior: melioration by stochastic synapses. J Neurosci. 2006;26: 3731–3744. doi: 10.1523/JNEUROSCI.5159-05.2006 16597727 PMC6674121

[82] Loewenstein Y, Seung HS. Operant matching is a generic outcome of synaptic plasticity based on the covariance between reward and neural activity. Proc Natl Acad Sci. 2006;103: 15224–15229. doi: 10.1073/pnas.0505220103 17008410 PMC1622804

[83] Simen P, Cohen JD. Explicit melioration by a neural diffusion model. Brain Res. 2009;1299: 95–117. doi: 10.1016/j.brainres.2009.07.017 19646968 PMC2763966

[84] Berg HC, Brown DA. Chemotaxis in Escherichia coli analysed by three-dimensional tracking. Nature. 1972;239: 500–504. doi: 10.1038/239500a0 4563019

[85] Sourjik V, Wingreen NS. Responding to chemical gradients: bacterial chemotaxis. Curr Opin Cell Biol. 2012;24: 262–268. doi: 10.1016/j.ceb.2011.11.008 22169400 PMC3320702

[86] Lazova MD, Ahmed T, Bellomo D, Stocker R, Shimizu TS. Response rescaling in bacterial chemotaxis. Proc Natl Acad Sci. 2011;108: 13870–13875. doi: 10.1073/pnas.1108608108 21808031 PMC3158140

[87] Berg HC. Random walks in biology. Expanded ed. Princeton, N.J: Princeton University Press; 1993.

[88] Si G, Wu T, Ouyang Q, Tu Y. Pathway-Based Mean-Field Model for Escherichia coli Chemotaxis. Phys Rev Lett. 2012;109: 048101. doi: 10.1103/PhysRevLett.109.048101 23006109 PMC3458579

[89] Dufour YS, Fu X, Hernandez-Nunez L, Emonet T. Limits of Feedback Control in Bacterial Chemotaxis. PLOS Comput Biol. 2014;10: e1003694. doi: 10.1371/journal.pcbi.1003694 24967937 PMC4072517

[90] Menolascina F, Rusconi R, Fernandez VI, Smriga S, Aminzare Z, Sontag ED, et al. Logarithmic sensing in Bacillus subtilis aerotaxis. NPJ Syst Biol Appl. 2017;3: 16036. doi: 10.1038/npjsba.2016.36 28725484 PMC5516866

[91] Keller EF, Segel LA. Model for chemotaxis. J Theor Biol. 1971;30: 225–234. doi: 10.1016/0022-5193(71)90050-6 4926701

[92] Roberts GO, Tweedie RL. Exponential convergence of Langevin distributions and their discrete approximations. Bernoulli. 1996;2: 341–363.

[93] Neal RM. MCMC using Hamiltonian dynamics. Handb Markov Chain Monte Carlo. 2011;2: 2.

[94] Girolami M, Calderhead B. Riemann manifold langevin and hamiltonian monte carlo methods. J R Stat Soc Ser B Stat Methodol. 2011;73: 123–214.

[95] Dalalyan AS. Theoretical guarantees for approximate sampling from smooth and log-concave densities. ArXiv Prepr ArXiv14127392. 2014.

[96] Sanchis-Segura C, Cline BH, Marsicano G, Lutz B, Spanagel R. Reduced sensitivity to reward in CB1 knockout mice. Psychopharmacology (Berl). 2004;176: 223–232. doi: 10.1007/s00213-004-1877-8 15083252

[97] Li X, Hoffman AF, Peng X-Q, Lupica CR, Gardner EL, Xi Z-X. Attenuation of basal and cocaine-enhanced locomotion and nucleus accumbens dopamine in cannabinoid CB1-receptor-knockout mice. Psychopharmacology (Berl). 2009;204: 1–11. doi: 10.1007/s00213-008-1432-0 19099297 PMC3729960

[98] Watabe-Uchida M, Eshel N, Uchida N. Neural circuitry of reward prediction error. Annu Rev Neurosci. 2017;40: 373–394. doi: 10.1146/annurev-neuro-072116-031109 28441114 PMC6721851

[99] Gös Ekman. Weber’s law and related functions. J Psychol. 1959;47: 343–352.

[100] Hart Y, Mayo AE, Shoval O, Alon U. Comparing apples and oranges: fold-change detection of multiple simultaneous inputs. PloS One. 2013;8: e57455. doi: 10.1371/journal.pone.0057455 23469195 PMC3587607

[101] Dabney W, Kurth-Nelson Z, Uchida N, Starkweather CK, Hassabis D, Munos R, et al. A distributional code for value in dopamine-based reinforcement learning. Nature. 2020;577: 671–675. doi: 10.1038/s41586-019-1924-6 31942076 PMC7476215

[102] Rothenhoefer KM, Hong T, Alikaya A, Stauffer WR. Rare rewards amplify dopamine responses. Nat Neurosci. 2021;24: 465–469. doi: 10.1038/s41593-021-00807-7 33686298 PMC9373731

[103] Salek MM, Carrara F, Fernandez V, Guasto JS, Stocker R. Bacterial chemotaxis in a microfluidic T-maze reveals strong phenotypic heterogeneity in chemotactic sensitivity. Nat Commun. 2019;10: 1877. doi: 10.1038/s41467-019-09521-2 31015402 PMC6478840

[104] Pierce-Shimomura JT, Morse TM, Lockery SR. The Fundamental Role of Pirouettes in Caenorhabditis elegans Chemotaxis. J Neurosci. 1999;19: 9557–9569. doi: 10.1523/JNEUROSCI.19-21-09557.1999 10531458 PMC6782915

[105] Polin M, Tuval I, Drescher K, Gollub JP, Goldstein RE. Chlamydomonas Swims with Two “Gears” in a Eukaryotic Version of Run-and-Tumble Locomotion. Science. 2009;325: 487–490. doi: 10.1126/science.1172667 19628868

[106] Luo L, Cook N, Venkatachalam V, Martinez-Velazquez LA, Zhang X, Calvo AC, et al. Bidirectional thermotaxis in Caenorhabditis elegans is mediated by distinct sensorimotor strategies driven by the AFD thermosensory neurons. Proc Natl Acad Sci U S A. 2014;111: 2776–2781. doi: 10.1073/pnas.1315205111 24550307 PMC3932917

[107] Kirkegaard JB, Bouillant A, Marron AO, Leptos KC, Goldstein RE. Aerotaxis in the closest relatives of animals. Elife. 2016;5: e18109. doi: 10.7554/eLife.18109 27882869 PMC5122458

[108] Hu B, Tu Y. Behaviors and strategies of bacterial navigation in chemical and nonchemical gradients. PLoS Comput Biol. 2014;10: e1003672. doi: 10.1371/journal.pcbi.1003672 24945282 PMC4063634

[109] Karin O, Alon U. Temporal fluctuations in chemotaxis gain implement a simulated-tempering strategy for efficient navigation in complex environments. Iscience. 2021;24: 102796. doi: 10.1016/j.isci.2021.102796 34345809 PMC8319753

[110] Gomperts SN, Kloosterman F, Wilson MA. VTA neurons coordinate with the hippocampal reactivation of spatial experience. Eichenbaum H, editor. eLife. 2015;4: e05360. doi: 10.7554/eLife.05360 26465113 PMC4695386

[111] Ólafsdóttir HF, Bush D, Barry C. The role of hippocampal replay in memory and planning. Curr Biol. 2018;28: R37–R50. doi: 10.1016/j.cub.2017.10.073 29316421 PMC5847173

[112] Stella F, Baracskay P, O’Neill J, Csicsvari J. Hippocampal reactivation of random trajectories resembling Brownian diffusion. Neuron. 2019;102: 450–461. doi: 10.1016/j.neuron.2019.01.052 30819547

[113] Lee AK, Wilson MA. Memory of sequential experience in the hippocampus during slow wave sleep. Neuron. 2002;36: 1183–1194. doi: 10.1016/s0896-6273(02)01096-6 12495631

[114] Pfeiffer BE, Foster DJ. Hippocampal place-cell sequences depict future paths to remembered goals. Nature. 2013;497: 74–79. doi: 10.1038/nature12112 23594744 PMC3990408

[115] Davidson TJ, Kloosterman F, Wilson MA. Hippocampal replay of extended experience. Neuron. 2009;63: 497–507. doi: 10.1016/j.neuron.2009.07.027 19709631 PMC4364032

[116] Chan F, Armstrong IT, Pari G, Riopelle RJ, Munoz DP. Deficits in saccadic eye-movement control in Parkinson’s disease. Neuropsychologia. 2005;43: 784–796. doi: 10.1016/j.neuropsychologia.2004.06.026 15721191

[117] Pretegiani E, Optican LM. Eye movements in Parkinson’s disease and inherited parkinsonian syndromes. Front Neurol. 2017;8: 592. doi: 10.3389/fneur.2017.00592 29170650 PMC5684125

[118] Sedaghat-Nejad E, Herzfeld DJ, Shadmehr R. Reward prediction error modulates saccade vigor. J Neurosci. 2019;39: 5010–5017. doi: 10.1523/JNEUROSCI.0432-19.2019 31015343 PMC6670245

[119] Stephen DG, Mirman D, Magnuson JS, Dixon JA. Lévy-like diffusion in eye movements during spoken-language comprehension. Phys Rev E. 2009;79: 056114. doi: 10.1103/PhysRevE.79.056114 19518528 PMC3694355

[120] Roberts JA, Wallis G, Breakspear M. Fixational eye movements during viewing of dynamic natural scenes. Front Psychol. 2013;4: 797. doi: 10.3389/fpsyg.2013.00797 24194727 PMC3810780

[121] Marlow CA, Viskontas IV, Matlin A, Boydston C, Boxer A, Taylor RP. Temporal structure of human gaze dynamics is invariant during free viewing. PloS One. 2015;10: e0139379. doi: 10.1371/journal.pone.0139379 26421613 PMC4589360

[122] Tsai H-C, Zhang F, Adamantidis A, Stuber GD, Bonci A, De Lecea L, et al. Phasic firing in dopaminergic neurons is sufficient for behavioral conditioning. Science. 2009;324: 1080–1084. doi: 10.1126/science.1168878 19389999 PMC5262197

[123] Chiang T-S, Hwang C-R, Sheu SJ. Diffusion for Global Optimization in $\mathbb{R}^n $. SIAM J Control Optim. 1987;25: 737–753. doi: 10.1137/0325042

[124] Gelfand SB, Mitter SK. Recursive Stochastic Algorithms for Global Optimization in $\mathbb{R}^d $. SIAM J Control Optim. 1991;29: 999–1018. doi: 10.1137/0329055

[125] Lee H, Risteski A, Ge R. Beyond Log-concavity: Provable Guarantees for Sampling Multi-modal Distributions using Simulated Tempering Langevin Monte Carlo. In: Bengio S, Wallach H, Larochelle H, Grauman K, Cesa-Bianchi N, Garnett R, editors. Advances in Neural Information Processing Systems 31. Curran Associates, Inc.; 2018. pp. 7847–7856. Available: http://papers.nips.cc/paper/8010-beyond-log-concavity-provable-guarantees-for-sampling-multi-modal-distributions-using-simulated-tempering-langevin-monte-carlo.pdf

[126] Erdogdu MA, Mackey L, Shamir O. Global Non-convex Optimization with Discretized Diffusions. In: Bengio S, Wallach H, Larochelle H, Grauman K, Cesa-Bianchi N, Garnett R, editors. Advances in Neural Information Processing Systems 31. Curran Associates, Inc.; 2018. pp. 9671–9680. Available: http://papers.nips.cc/paper/8175-global-non-convex-optimization-with-discretized-diffusions.pdf

[127] Ma Y-A, Chen Y, Jin C, Flammarion N, Jordan MI. Sampling can be faster than optimization. Proc Natl Acad Sci. 2019;116: 20881–20885. doi: 10.1073/pnas.1820003116 31570618 PMC6800351

[128] Chen Y, Chen J, Dong J, Peng J, Wang Z. Accelerating Nonconvex Learning via Replica Exchange Langevin Diffusion. ArXiv200701990 Cs Math Stat. 2020 [cited 13 Oct 2020]. Available: http://arxiv.org/abs/2007.01990

[129] Long J, Zucker SW, Emonet T. Feedback between motion and sensation provides nonlinear boost in run-and-tumble navigation. PLoS Comput Biol. 2017;13: e1005429. doi: 10.1371/journal.pcbi.1005429 28264023 PMC5358899

[130] Eisenegger C, Naef M, Linssen A, Clark L, Gandamaneni PK, Müller U, et al. Role of dopamine D2 receptors in human reinforcement learning. Neuropsychopharmacology. 2014;39: 2366–2375. doi: 10.1038/npp.2014.84 24713613 PMC4138746

[131] Cinotti F, Fresno V, Aklil N, Coutureau E, Girard B, Marchand AR, et al. Dopamine blockade impairs the exploration-exploitation trade-off in rats. Sci Rep. 2019;9: 1–14. 31043685 10.1038/s41598-019-43245-z PMC6494917

[132] Frank MJ, Doll BB, Oas-Terpstra J, Moreno F. The neurogenetics of exploration and exploitation: Prefrontal and striatal dopaminergic components. Nat Neurosci. 2009;12: 1062. 19620978 10.1038/nn.2342 PMC3062477

[133] Costa VD, Tran VL, Turchi J, Averbeck BB. Dopamine modulates novelty seeking behavior during decision making. Behav Neurosci. 2014;128: 556. doi: 10.1037/a0037128 24911320 PMC5861725

[134] Raginsky M, Rakhlin A, Telgarsky M. Non-convex learning via Stochastic Gradient Langevin Dynamics: a nonasymptotic analysis. ArXiv170203849 Cs Math Stat. 2017 [cited 2 Aug 2020]. Available: http://arxiv.org/abs/1702.03849

[135] Xu P, Chen J, Zou D, Gu Q. Global Convergence of Langevin Dynamics Based Algorithms for Nonconvex Optimization. In: Bengio S, Wallach H, Larochelle H, Grauman K, Cesa-Bianchi N, Garnett R, editors. Advances in Neural Information Processing Systems 31. Curran Associates, Inc.; 2018. pp. 3122–3133. Available: http://papers.nips.cc/paper/7575-global-convergence-of-langevin-dynamics-based-algorithms-for-nonconvex-optimization.pdf

[136] Barkai N, Leibler S. Robustness in simple biochemical networks. Nature. 1997;387: 913–917. doi: 10.1038/43199 9202124

[137] Alon U, Surette MG, Barkai N, Leibler S. Robustness in bacterial chemotaxis. Nature. 1999;397: 168–171. doi: 10.1038/16483 9923680

[138] Ferrell JE Jr. Perfect and near-perfect adaptation in cell signaling. Cell Syst. 2016;2: 62–67. doi: 10.1016/j.cels.2016.02.006 27135159

[139] Stauffer WR, Lak A, Schultz W. Dopamine reward prediction error responses reflect marginal utility. Curr Biol. 2014;24: 2491–2500. doi: 10.1016/j.cub.2014.08.064 25283778 PMC4228052

[140] Doya K. Reinforcement learning in continuous time and space. Neural Comput. 2000;12: 219–245. doi: 10.1162/089976600300015961 10636940

[141] Barto AG, Sutton RS, Watkins C. Learning and sequential decision making. University of Massachusetts Amherst, MA; 1989.

[142] Coulthard EJ, Bogacz R, Javed S, Mooney LK, Murphy G, Keeley S, et al. Distinct roles of dopamine and subthalamic nucleus in learning and probabilistic decision making. Brain. 2012;135: 3721–3734. doi: 10.1093/brain/aws273 23114368 PMC3525052

