# Supplementary material for: The dopamine circuit as a reward-taxis navigation system
Source: PLoS Comput Biol. 2022 Jul 25;18(7):e1010340. doi: 10.1371/journal.pcbi.1010340 (PMC9352198; doi:10.1371/journal.pcbi.1010340)
Supplement: S1 Text — (DOCX) [file pcbi.1010340.s001.docx]

**Supplementary Theory**

**The dopamine circuit as a reward-taxis navigation system**

Karin et al.

**Derivation of Langevin dynamics and matching law from run-and-tumble model.**

Here we derive the Langevin dynamics for dopaminergic-driven navigation, which will be given by:

$$dx=\chi\nabla\log R(x)dt+\sqrt{2D} dW [ SEQ Eq \backslash* MERGEFORMAT 1]$$

where $W$ is an *m*-dimensional Wiener process and $R(x)$ is the expected reward field. The advection term is $\chi=m^{-1}v^{2}\tau\cdot\mu d_{0}^{-1}$, where *m* is the dimension, *v* is the movement speed of the animal,$\mu$ is the dopaminergic gain, and $d_{0}$ is the adapted activity of the dopaminergic neurons. The diffusion term is $D=m^{-1}v^{2}\tau$. We first consider a model where reorientation frequency $\phi^{-1}$ is inhibited by dopamine: $\phi=\tau\left( \frac{d}{d_{0}} \right)$, and in the next section consider a model where dopamine controls movement speed.

The derivation largely follows the derivation of spatio-temporal dynamics of chemotaxis by Si et al. [1], which is provided here for completeness. We will derive here the one-dimensional case (we confirmed the 2D case using numerical simulations). In order to derive the stochastic dynamics of an individual animal, we construct a Fokker-Planck equation for the probability density $P^{\pm}(g,x,t)$ that denotes the probability that an animal with GABAergic activation $g$ is at location *x* at time *t*, moving at direction $\pm.$ We denote the reorientation frequency as $z=\phi^{-1}$. The master equation for $P^{\pm}(g,x,t)$ includes changes due to movement in space, changes in GABA activation, and reorientations:

$$\frac{\partial P^{+}\left( g,x,t \right)}{\partial t}=-\frac{\partial\left( vP^{+} \right)}{\partial x}-\frac{\partial\left( \dot{g}P^{+} \right)}{\partial g}-\frac{z(g)}{2}\left( P^{+}-P^{-} \right) [ SEQ Eq \backslash* MERGEFORMAT 2]$$

$$\frac{\partial P^{-}\left( g,x,t \right)}{\partial t}=\frac{\partial\left( vP^{-} \right)}{\partial x}-\frac{\partial\left( \dot{g}P^{-} \right)}{\partial g}+\frac{z(g)}{2}\left( P^{+}-P^{-} \right) [ SEQ Eq \backslash* MERGEFORMAT 3]$$

To simplify the analysis, Si et al. approximate $P^{\pm}$ as a function of the mean methylation level. In the dopamine context, this corresponds to averaging over GABAergic activity *g*, i.e. assuming that all individuals have GABAergic activation level $G^{\pm}(x,t)$ (we then take $\phi$ to be the run duration at that GABA level). Another simplifying assumption is that due to rapid tumbling, the difference between GABA levels $\Delta G\left( x,t \right)=\frac{1}{2}\left( G^{+}\left( x,t \right)-G^{-}\left( x,t \right) \right)$ can be approximated by the difference in average GABA level over the run length:

$$\Delta G\approx-\frac{\partial G}{\partial x}Z^{-1}v [ SEQ Eq \backslash* MERGEFORMAT 4]$$

Where $G\left( x,t \right)=\frac{P_{G}^{+}G^{+}+P_{G}^{-}G^{-}}{P_{G}^{+}+P_{G}^{-}}$is the population-weighted average of GABA activation, and $P_{G}^{+},P_{G}^{-}$ correspond to the probability densities integrated over *g*, and we denote $z\left( G \right)=Z$.

In order to derive the Fokker-Planck equation, we define density and flux:

$$\rho\left( x,t \right)=\int\left[ P^{+}+P^{-} \right]dg [ SEQ Eq \backslash* MERGEFORMAT 5]$$

$$J\left( x \right)=\int\left[ v\left( P^{+}-P^{-} \right) \right]dg [ SEQ Eq \backslash* MERGEFORMAT 6]$$

Adding Eqs. $2$, $3$ and integrating over *g* we get:

$$\frac{\partial\rho}{\partial t}=-\frac{\partial J}{\partial x} [ SEQ Eq \backslash* MERGEFORMAT 7]$$

Subtracting Eqs. $2$, $3$, multiplying by *v*, and integrating over *g* we get:

$$\frac{\partial J}{\partial t}=-v^{2}\int\frac{\partial}{\partial x}\left( P^{+}+P^{-} \right)dg-v\int\left( z\left( g \right)P^{+}- z\left( g \right)P^{-} \right)dg=-v^{2}\frac{\partial}{\partial x}\rho-v\left( z\left( G^{+} \right)P_{G}^{+}- z\left( G^{-} \right)P_{G}^{-} \right) [ SEQ Eq \backslash* MERGEFORMAT 8]$$

Linearizing over $G:$

$$z(G^{\pm})\approx z(G)+\frac{\partial Z}{\partial G}(G^{\pm}-G) [ SEQ Eq \backslash* MERGEFORMAT 9]$$

We get:

$$z\left( G^{+} \right)P_{G}^{+}- z\left( G^{-} \right)P_{G}^{-}=z\left( G \right)\left( P_{G}^{+}-P_{G}^{-} \right)+\frac{\partial Z}{\partial G}\left( \left( G^{+}-G \right)P_{G}^{+}-\left( G^{-}-G \right)P_{G}^{-} \right)=\frac{ZJ}{v}+\frac{\partial Z}{\partial G}\left( \frac{2\left( G^{+}-G^{-} \right)}{\rho}\left( P_{G}^{+}P_{G}^{-} \right) \right)=\frac{ZJ}{v}+\frac{\partial Z}{\partial G}\left( \frac{\frac{1}{2}\left( G^{+}-G^{-} \right)}{\rho}\left( \rho^{2}-\frac{J^{2}}{v^{2}} \right) \right)=\frac{ZJ}{v}+\frac{\partial Z}{\partial G}\left( \Delta G \rho\left( 1-\frac{J^{2}}{{\rho^{2}v}^{2}} \right) \right) [ SEQ Eq \backslash* MERGEFORMAT 10]$$

We neglect the term $\frac{J^{2}}{{\rho^{2}v}^{2}}\ll1$. Plugging Eq. $10$ into Eq. $8$, we get:

$$\frac{\partial J}{\partial t}=-v^{2}\frac{\partial}{\partial x}\rho-ZJ-v\frac{\partial Z}{\partial G}\Delta G \rho[ SEQ Eq \backslash* MERGEFORMAT 11]$$

Taking $\frac{\partial J}{\partial t}=0$, we get that:

$$J=-v^{2}Z^{-1}\frac{\partial}{\partial x}\rho-vZ^{-1}\frac{\partial Z}{\partial G}\Delta G\rho\approx-v^{2}Z^{-1}\frac{\partial\rho}{\partial x}-v^{2}\frac{\partial Z^{-1}}{\partial G}\frac{\partial G}{\partial x}\rho[ SEQ Eq \backslash* MERGEFORMAT 12]$$

The final equation is for *G*, which is derived by adding Eqs. $2$, $3$ multiplying by *g* and integrating over *g*:

$$\int\frac{\partial P^{+}g}{\partial t}+\frac{\partial P^{-}g}{\partial t}dg=-\int g\left( \frac{\partial\left( vP^{+} \right)}{\partial x}+\frac{\partial\left( vP^{-} \right)}{\partial x} \right)dg-\int g\left( \frac{\partial\left( \dot{g}P^{+} \right)}{\partial g}+\frac{\partial\left( \dot{g}P^{-} \right)}{\partial g} \right)dg [ SEQ Eq \backslash* MERGEFORMAT 13]$$

The left-hand side is simply:

$$\int\frac{\partial P^{+}g}{\partial t}+\frac{\partial P^{-}g}{\partial t}dg=\frac{\partial}{\partial t}\left( P_{G}^{+}G^{+}+P_{G}^{-}G^{-} \right)=\frac{\partial}{\partial t}\rho G=\frac{\partial G}{\partial t}\rho+\frac{\partial\rho}{\partial t}G [ SEQ Eq \backslash* MERGEFORMAT 14]$$

For the right-hand side:

$$-\int g\left( \frac{\partial\left( vP^{+} \right)}{\partial x}-\frac{\partial\left( vP^{-} \right)}{\partial x} \right)dg=-\frac{\partial}{\partial x}v\left( P_{G}^{+}G^{+}-P_{G}^{-}G^{-} \right)==-\frac{\partial}{\partial x}v\left( P_{G}^{+}G+P_{G}^{+}\left( G^{+}-G \right)-P_{G}^{-}G-P_{G}^{-}\left( G^{-}-G \right) \right)=-\frac{\partial}{\partial x}\left( JG+v\Delta G\rho\left( 1-\frac{J^{2}}{{\rho^{2}v}^{2}} \right) \right)\approx-\frac{\partial}{\partial x}\left( JG+v\Delta G\rho\right)=\frac{\partial\rho}{\partial t}G-\frac{\partial G}{\partial x}J-\frac{\partial}{\partial x}v\Delta G\rho[ SEQ Eq \backslash* MERGEFORMAT 15]$$

And:

$$-\int g\left( \frac{\partial\left( \dot{g}P^{+} \right)}{\partial g}+\frac{\partial\left( \dot{g}P^{-} \right)}{\partial g} \right)dg=\left( \dot{g}\left( G^{+} \right)P_{G}^{+}+\dot{g}\left( G^{-} \right)P_{G}^{-} \right)\approx\dot{g}\rho[ SEQ Eq \backslash* MERGEFORMAT 16]$$

So we get:

$$\frac{\partial G}{\partial t}\rho+\frac{\partial\rho}{\partial t}G=\dot{g}\rho+\frac{\partial\rho}{\partial t}G-\frac{\partial G}{\partial x}J-\frac{\partial}{\partial x}v\Delta G\rho$$

$$\frac{\partial G}{\partial t}=\dot{g}-\frac{\partial G}{\partial x}J\rho^{-1} -\frac{1}{\rho}\left( \frac{\partial}{\partial x}v\Delta G \right) [ SEQ Eq \backslash* MERGEFORMAT 17]$$

It is assumed that the first term dominates Eq. $17$, so dopamine level is close to being adapted $d\approx d_{0}$ everywhere. This gives $\frac{\partial G}{\partial x}\approx\alpha^{-1}\mu\nabla\log R$, and $\frac{\partial\phi}{\partial G}=-\frac{\tau\alpha}{d_{0}}$ , and $Z^{-1}\approx\tau$, so that, by combining Eqs., $7$, $12$, we get:

$$\frac{\partial\rho}{\partial t}\approx\frac{\partial}{\partial x}\left( v^{2}Z^{-1}\frac{\partial\rho}{\partial x}+v^{2}\frac{\partial Z^{-1}}{\partial G}\frac{\partial G}{\partial x}\rho\right)=v^{2}\tau\left( \frac{\partial}{\partial x}\frac{\partial\rho}{\partial x}-\frac{\partial}{\partial x}\frac{\mu}{d0}\nabla\log R\rho\right) [ SEQ Eq \backslash* MERGEFORMAT 18]$$

This is the Fokker-Planck equation for the evolution of the probability density of the location of the animal over time, which directly corresponds to Eq. 1 and from which the generalized matching law can be derived.

In the minimal model we considered the case where movement direction was chosen at random after each reorientation. This assumption can be relaxed by allowing for persistence in movement direction after reorientations, or more generally allowing for correlations between the movement direction before and after a reorientation event. Such directional persistence is, in fact, common in run-and-tumble unicellular navigation. While E. coli tumbles result in reorientation angles that are only mildly correlated with the original swimming direction, the reorientations of choanoflagellates only slightly shift their swimming direction [2,3]. The effects of directional persistence have been extensively modeled in the literature [4–6]. Roughly, they extend the average run duration $\tau$. Since in our model, changing $\tau$ increases proportionally both the drift term $\chi$ and the diffusion term $\sigma$ in Eq. 1, as long as $\tau$ remains fast compared with the adaptation timescale. This does not affect the conclusions of this work, including the derived matching law, as we verified by numerical simulations.

**Derivation of Langevin dynamics and matching law from model where dopamine controls movement speed.**

Here we will analyze a model where the reorientation/tumble rate is constant ($z^{-1}=\phi=\tau$) and dopamine controls movement speed: $v\left( d \right)=v_{0}\frac{d}{d_{0}}$. We will show that the average dynamics, approximated by Eq. 1, are the same for this model, suggesting that our conclusions generalize to the case where dopamine controls movement speed.

The master equation for this model is:

$$\frac{\partial P^{+}\left( g,x,t \right)}{\partial t}=-\frac{\partial\left( v(g)P^{+} \right)}{\partial x}-\frac{\partial\left( \dot{g}P^{+} \right)}{\partial g}-\frac{\tau^{-1}}{2}\left( P^{+}-P^{-} \right) [ SEQ Eq \backslash* MERGEFORMAT 19]$$

$$\frac{\partial P^{-}\left( g,x,t \right)}{\partial t}=\frac{\partial\left( v(g)P^{-} \right)}{\partial x}-\frac{\partial\left( \dot{g}P^{-} \right)}{\partial g}+\frac{\tau^{-1}}{2}\left( P^{+}-P^{-} \right) [ SEQ Eq \backslash* MERGEFORMAT 20]$$

We again define:

$$\rho\left( x,t \right)=\int\left[ P^{+}+P^{-} \right]dg [ SEQ Eq \backslash* MERGEFORMAT 21]$$

And:

$$Q\left( x,t \right)=\int\left[ P^{+}-P^{-} \right]dg [ SEQ Eq \backslash* MERGEFORMAT 22]$$

We will again take the mean field assumption that $P^{\pm}\left( g,x,t \right)=P_{G}^{\pm}\left( x,t \right)\delta\left( g-G^{\pm}\left( x,t \right) \right)$, with $G^{+},G^{-}$ as the levels of GABA activation of individuals moving in the $+,-$ direction, with the corresponding movement speeds $V^{+},V^{-}$. Adding the equations and integrating over *g*:

$$\frac{\partial\rho}{\partial t}=-\int\left( \frac{\partial\left( v\left( g \right)P^{+} \right)}{\partial x}-\frac{\partial\left( v\left( g \right)P^{-} \right)}{\partial x} \right)dg=-\frac{\partial}{\partial x}\left( V^{+}P_{G}^{+}-V^{-}P_{G}^{-} \right) [ SEQ Eq \backslash* MERGEFORMAT 23]$$

Linearizing around the population-weighted average $G$:

$$V^{\pm}\approx V+\frac{\partial V}{\partial G}(G^{\pm}-G) [ SEQ Eq \backslash* MERGEFORMAT 24]$$

We get:

$$\frac{\partial\rho}{\partial t}=-\frac{\partial}{\partial x}\left( VQ+\frac{\partial V}{\partial G}\left( \left( G^{+}-G \right)P_{G}^{+}-\left( G^{-}-G \right)P_{G}^{-} \right) \right)=-\frac{\partial}{\partial x}\left( VQ+\frac{\partial V}{\partial G}\Delta G\rho\left( 1-\frac{Q^{2}}{\rho^{2}} \right) \right)\approx-\frac{\partial}{\partial x}\left( VQ+\frac{\partial V}{\partial G}\Delta G\rho\right) [ SEQ Eq \backslash* MERGEFORMAT 25]$$

Subtracting the equations and integrating over *g* provides:

$$\frac{\partial Q}{\partial t}=-\int\left( \frac{\partial\left( v\left( g \right)P^{+} \right)}{\partial x}+\frac{\partial\left( v\left( g \right)P^{-} \right)}{\partial x} \right)dg-\tau^{-1}Q=-\frac{\partial}{\partial x}\left( V^{+}P_{G}^{+}+V^{-}P_{G}^{-} \right)-\tau^{-1}Q [ SEQ Eq \backslash* MERGEFORMAT 26]$$

Since we are interested in timescales that go beyond the tumbling rate, we can take the left-hand side of the equation to be zero, so we get:

$$Q=-\tau\frac{\partial}{\partial x}\left( V^{+}P_{G}^{+}+V^{-}P_{G}^{-} \right)=-\tau\frac{\partial}{\partial x}V\rho[ SEQ Eq \backslash* MERGEFORMAT 27]$$

Where $V\left( x,t \right)=\frac{P_{G}^{+}V^{+}+P_{G}^{-}G^{-}}{P_{G}^{+}+P_{G}^{-}}$. As before, we will assume that the dynamics are governed by adaptation, so $V\approx v_{0}$.

Plugging this into Eq. $25$ gives:

$$\frac{\partial\rho}{\partial t}=-\frac{\partial}{\partial x}\left( -\tau v_{0}^{2}\frac{\partial\rho}{\partial x}+\frac{\partial V}{\partial G}\Delta G\rho\right) [ SEQ Eq \backslash* MERGEFORMAT 28]$$

Using the approximation $\Delta G\approx-\frac{\partial G}{\partial x}\tau V$, we get that:

$$\frac{\partial\rho}{\partial t}=-\frac{\partial}{\partial x}\left( -\tau v_{0}^{2}\frac{\partial\rho}{\partial x}-\tau\frac{\partial V}{\partial G}\frac{\partial G}{\partial x}\rho v_{0} \right) [ SEQ Eq \backslash* MERGEFORMAT 29]$$

Assuming that dopamine level is close to being adapted $d\approx d_{0}$, we have $\frac{\partial G}{\partial x}\approx\alpha^{-1}\mu\nabla\log r$, and $\frac{\partial V}{\partial G}=-\frac{v_{0}\alpha}{d0}$ , so that:

$$\frac{\partial\rho}{\partial t}=v_{0}^{2}\tau\left( \frac{\partial}{\partial x}\frac{\partial\rho}{\partial x}-\frac{\partial}{\partial x}\frac{\mu}{d0} \nabla\log r\rho\right) [ SEQ Eq \backslash* MERGEFORMAT 30]$$

This equation is the same as the dynamics for the case where dopamine controls run duration, suggesting that the average behavior is the same whether dopamine controls movement speed or run duration.

**Further generalizations of the run-and-tumble model.**

Here we test several other generalizations of the run-and-tumble model, and show that they do not affect our main conclusions. Consider an animal performing run-and-tumble navigation in an environment where the expected reward is *R*, and *d* is an internal variable that determines run duration $\phi(d)$ and that adapts to a baseline *d_0_*. In our case, *d* is dopamine and adaptation to *d_0_* is due to GABAergic inhibition. The invariant distribution is [7]:

$$P\left( x \right)\approx\frac{\Omega}{v}e^{\int_{-\infty}^{x} \left( \frac{\partial\log\phi}{\partial d} \frac{\partial d}{\partial R} \right)_{d=d_{0}}dR} [ SEQ Eq \backslash* MERGEFORMAT 31]$$

Where $\Omega$ is a normalization constant, we assume that tumbling is rapid relative to the adaptation of *d*, and we assume that dopamine *d* is on average close to $d_{0}$ (see Hu & Tu for derivation details).

Using Eq. $31$, we can derive general conditions for which *d*, $\phi$ can provide the general matching law, which requires an invariant distribution such that $\frac{P\left( x_{1} \right)}{P\left( x_{2} \right)}=\left( \frac{r_{1}}{r_{2}} \right)^{\beta}$ for rewards of magnitude $r_{1},r_{2}$ at locations $x_{1},x_{2}$, i.e.:

$$\left( \frac{r_{1}}{r_{2}} \right)^{\beta}=\frac{P\left( x_{1} \right)}{P\left( x_{2} \right)}=e^{\int_{-\infty}^{x_{1}} \left( \frac{\partial\log\phi}{\partial d} \frac{\partial d}{\partial R} \right)_{d=d_{0}}dR-\int_{-\infty}^{x_{2}} \left( \frac{\partial\log\phi}{\partial d} \frac{\partial d}{\partial R} \right)_{d=d_{0}}dR} [ SEQ Eq \backslash* MERGEFORMAT 32]$$

One can see that if dopamine gates the run duration: $\phi\propto\phi_{0}d$ then $\left( \frac{\partial\log\phi}{\partial d} \right)_{d=d_{0}}=\frac{1}{d_{0}}$, and if dopamine is activated by the logarithm of reward: $d=\mu\log R+C$, then $\frac{\partial d}{\partial R}=\frac{\mu}{R}$ and from Eq. $32$:

$$\frac{P\left( x_{1} \right)}{P\left( x_{2} \right)}=e^{\frac{\mu}{d_{0}}\log r_{1}/r_{2}}=\left( \frac{r_{1}}{r_{2}} \right)^{\frac{\mu}{d_{0}}} [ SEQ Eq \backslash* MERGEFORMAT 33]$$

Providing the matching law with $\beta=\frac{\mu}{d_{0}}$. It is important to notice that the precise circuit architecture (feedforward or feedback) is not important, only the logarithmic activation of dopamine, and adaptation.

Other possible circuit architectures do not provide the matching law. If dopamine is directly activated by reward *R*, i.e., $d=\mu R^{\alpha}+D$ (with $\alpha>0$), then $\frac{\partial d}{\partial R}=\alpha\mu R^{\alpha-1}$ and from Eq. $32$:

$$\frac{P\left( x_{1} \right)}{P\left( x_{2} \right)}=e^{\frac{\mu}{d_{0}}\left( r_{1}^{\alpha}-r_{2}^{\alpha} \right)} [ SEQ Eq \backslash* MERGEFORMAT 34]$$

Which does not provide the matching law. Another possibility is that dopamine (and hence reorientation frequency) depends on *R*, rather than a gradient of *R*. This possibility, which was analyzed for chemotaxis by Schnitzer [4], results in a uniform (i.e. reward independent) stationary distribution and thus cannot explain the matching law. Finally, movement speed *v* that depends on the spatial position or on the reward can lead to accumulation near rewards, however to provide a positive relation: $R\left( x_{1} \right)>R\left( x_{2} \right)\to P\left( x_{1} \right)>P(x_{2})$, it is necessary that running speed decreases with dopamine, which is in contrast with the invigorating effect of dopamine on motion.

**Reference**

1. Si G, Wu T, Ouyang Q, Tu Y. Pathway-Based Mean-Field Model for Escherichia coli Chemotaxis. Phys Rev Lett. 2012;109: 048101. doi:10.1103/PhysRevLett.109.048101

2. Berg HC, Brown DA. Chemotaxis in Escherichia coli analysed by three-dimensional tracking. Nature. 1972;239: 500–504.

3. Kirkegaard JB, Bouillant A, Marron AO, Leptos KC, Goldstein RE. Aerotaxis in the closest relatives of animals. Elife. 2016;5: e18109.

4. Schnitzer MJ. Theory of continuum random walks and application to chemotaxis. Phys Rev E. 1993;48: 2553.

5. Locsei JT. Persistence of direction increases the drift velocity of run and tumble chemotaxis. J Math Biol. 2007;55: 41–60.

6. Kirkegaard JB, Goldstein RE. The role of tumbling frequency and persistence in optimal run-and-tumble chemotaxis. IMA J Appl Math. 2018;83: 700–719.

7. Hu B, Tu Y. Behaviors and strategies of bacterial navigation in chemical and nonchemical gradients. PLoS Comput Biol. 2014;10: e1003672.
