# Supplementary figures and images for: The dopamine circuit as a reward-taxis navigation system
Source: PLoS Comput Biol. 2022 Jul 25;18(7):e1010340. doi: 10.1371/journal.pcbi.1010340 (PMC9352198; doi:10.1371/journal.pcbi.1010340)

**S1 Fig.**

**
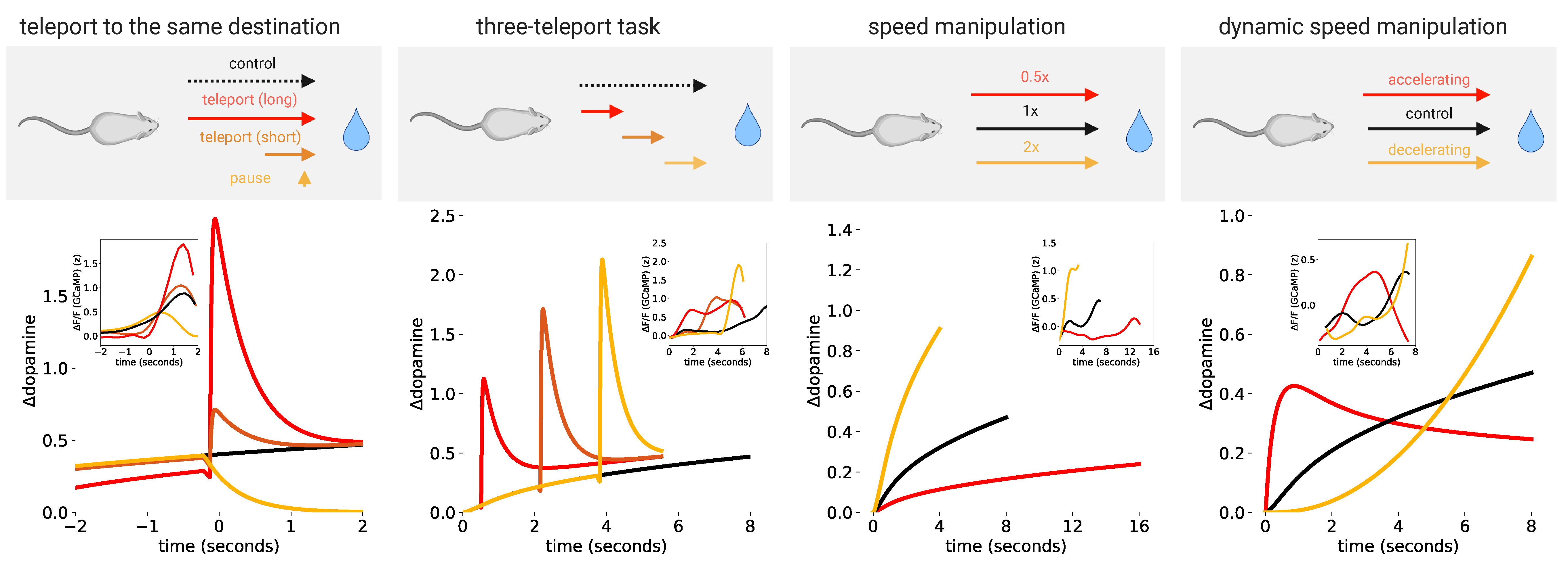
**

Supplement: S1 Fig — Dopamine output to movement in a reward gradient given by R(x)=e−γxh, with h = 1.5, x0 = 1, v0 = 1 and γ = 0.04, and perturbations as described in Fig 2 of [12]. Insets. Corresponding dopaminergic outputs from mice (left to right: n = 11, n = 11, n = 15, n = 5) VTA neurons measured by calcium imaging, from Fig 2C, 2G, 2K and 2O in [12], smoothed using a Savitzky–Golay filter. All simulations were performed with the parameters provided in Table 1. (DOCX) [file pcbi.1010340.s002.docx]

**S3 Fig.**

**
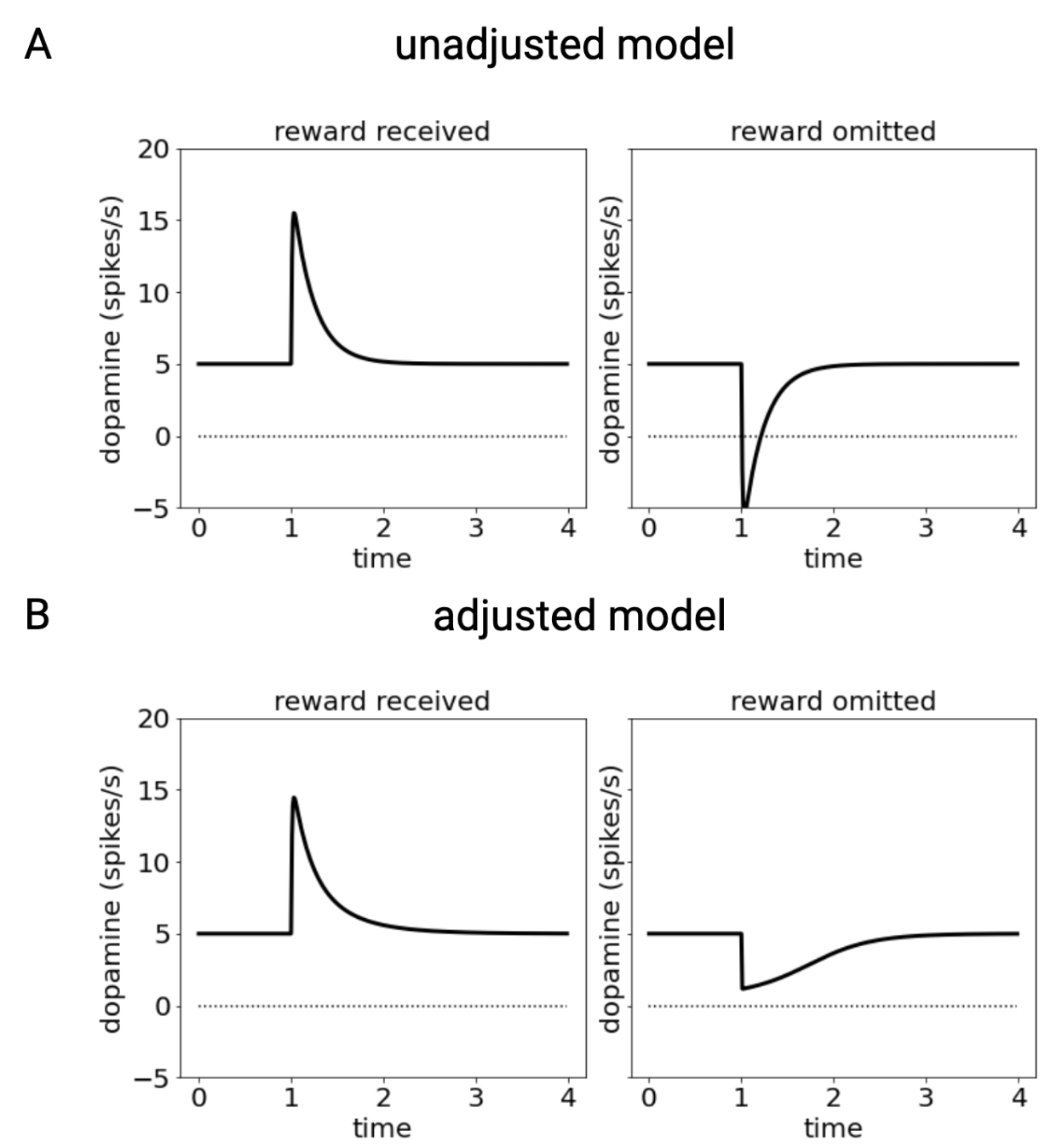
**

Supplement: S3 Fig — (A) Reward reception and omission was simulated according to Eqs 1,2 in the manuscript in a manner similar to the simulations in Fig 1. The input is given by R(t) = R0+λθ(t−t0) where θ(t−t0) is a unit step function, R0 = 1 and λ = 7 for the reward reception and R0 = 7, λ = −6 for reward omission. Note that Eq 1 reaches negative values upon reward omission. (B) Dynamics for model where Eq 1 is adjusted as d=C+μlogR−αgdd+kd (here taking kd = 1). This model does not reach negative values of d, and behaves similarly to the FCD model if kd≪d0. (DOCX) [file pcbi.1010340.s004.docx]

**S4 Fig.**

**
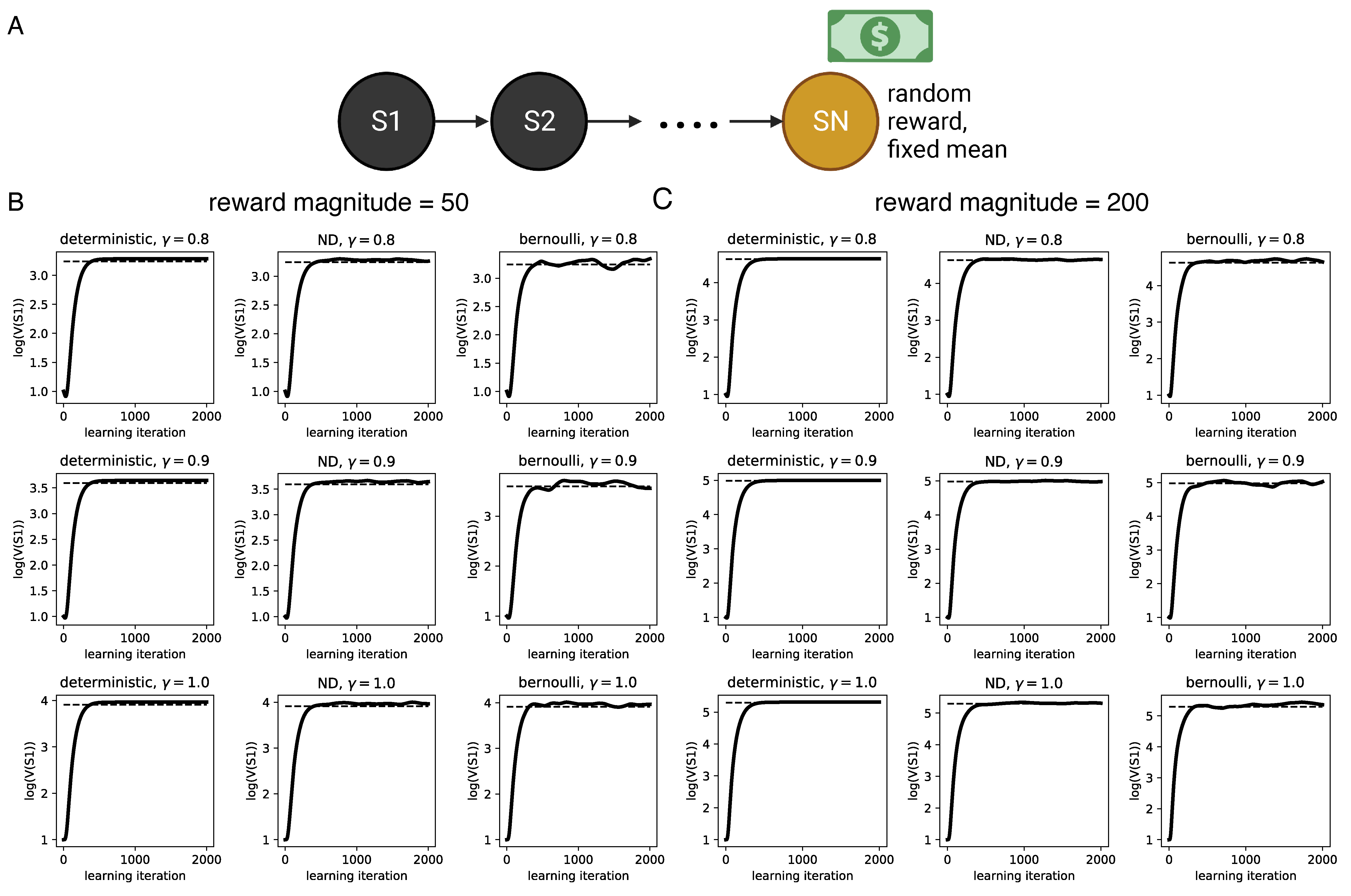
**

Supplement: S4 Fig — (A) Simulation setup. The agent progresses through a series of N states S1,…,SN, where in the final stage SN a reward is drawn according to a distribution with a fixed mean reward value. In the simulations we use three distributions (deterministic reward, normal distribution with CV = 0.3, and Bernoulli trials). A logarithmic value function log V is learned recursively according to the rule logVt+1(s)←logVt(s)+α(elog(r(t)+γVt(s+1))−logVt(s)−1). (B,C) Learning simulations with reward magnitude 50 (B) and 200 (C). Thick line denotes a log Vt+1(S1) in a single simulation, while thin dashed line denotes expected logVt+1(s)=logE[∑t=t0∞γtr(t)dt]. Simulation parameters are N = 5, α = 0.02. Figures were created with BioRender.com. (DOCX) [file pcbi.1010340.s005.docx]
