# Supplementary material for: The dopamine circuit as a reward-taxis navigation system
Source: PLoS Comput Biol. 2022 Jul 25;18(7):e1010340. doi: 10.1371/journal.pcbi.1010340 (PMC9352198; doi:10.1371/journal.pcbi.1010340)
Supplement: Attachment — Submitted filename: rebuttal_.docx [file pcbi.1010340.s006.docx]

**Dear Editors,**

**Thank you for the positive consideration of our manuscript and for the referee reports. We now edited to manuscript according to the suggestions of the reviewers, including adding relevant discussion sections and clarifying the text as suggested.**

**Yours,**

**Omer Karin and Uri Alon**

Reviewer #1: In this paper, the authors show that modeling dopamine release as a response to fold changes in expected reward, together with changes in organism motion with dopaminergic neuron firing rates, can produce “reward-taxis” and explain several experimental observations. These include: how the maximum change in neuron firing rates with changes in reward size (Fig 2B), response rescaling when a cue is provided ahead of a reward (Fig 2DE), and matching laws of operant behavior (Fig 3). The authors draw an intriguing analogy to bacterial chemotaxis.

I find the idea interesting and compelling, but I have some comments.

**We thank the reviewer for this endorsement of our work.**

My main comment is that a logical error is made around line 318 and after: while I agree that \beta should equal \chi/D, the functional form of \chi is highly dependent on the specific ways in which dopamine responses alter animal behavior. Therefore, \beta = \mu/d0 is only true if one assumes the authors’ simple model for how dopamine elicits changes in behavior, and one cannot in general estimate \beta from \mu and d0. You need to know the details of the system to determine an expression for \chi and thus \beta. The coincidental agreement between \beta and \mu/do in one system (around Line 323) is therefore not convincing.

**We agree with the reviewer that the value of** $\boldsymbol{\chi}$ **depends on the regulation of behavior by dopamine. Under our assumptions – that movement speed or reorientation frequency in is proportional to dopamine levels – we obtain the relationship of** $\boldsymbol{\beta=}\frac{\boldsymbol{\mu}}{\boldsymbol{d}_{\boldsymbol{0}}}$**. While these assumptions are motivated by empirical observations, we agree that more precise experimental characterization is needed to obtain a more accurate estimate of** $\boldsymbol{\chi}$**. For example, nonlinear gain in the regulation of movement by dopamine (**$\boldsymbol{v\propto}\boldsymbol{d}^{\boldsymbol{h}}$**) would lead to multiplicative prefactor** $\boldsymbol{h}$ **in** $\boldsymbol{\beta}$**; our estimate will then be accurate only if these nonlinearities are mild. Additionally, longer runs relative to the adaptation timescale can also affect** $\boldsymbol{\beta}$**. Again, as long as these assumptions are reasonably met (runs are not much longer than the adaptation time), we expect that our estimate of** $\boldsymbol{\beta\approx1}$ **should still hold, however, these are important caveats that we now include in our discussion (Lines 456-465):**

***The stochastic model is sufficient for explaining matching behavior, and provides an accurate mechanistic estimate for the matching constant*** $\boldsymbol{\beta}$***. The estimate is derived under mild mechanistic assumptions – that movement speed (or run length) is controlled proportionally by dopamine levels, and that run times are relatively short compared with adaptation time. Improved experimental characterization of movement control by dopamine will allow us to relax these assumptions to get a better estimate for*** $\boldsymbol{\beta}$***. For example, a nonlinear gain for movement speed regulation by dopamine*** $\boldsymbol{v\propto}\boldsymbol{d}^{\boldsymbol{h}}$ ***would result in multiplication by a constant prefactor*** $\boldsymbol{\beta}\boldsymbol{=h\mu/}\boldsymbol{d}_{\boldsymbol{0}}$***, while longer run times would result in a proportional decrease in*** $\boldsymbol{\beta}$ ***(101). As long as these effects are mild, we expect our estimate*** $\boldsymbol{\beta\approx1}$ ***to hold. Importantly, we still expect the matching constant*** $\boldsymbol{\beta}$ ***to be proportional to*** $\boldsymbol{\mu}$ ***and inversely proportional to*** $\boldsymbol{d}_{\boldsymbol{0}}$***.***

Some explanations throughout could be clearer. Line 121, it would be helpful to explicitly indicate which quantities depend on time.

**We now fixed this in the text.**

The explanations around Line 184 are confusing because they refer to both R and u, but R is just a linear function of u. To explain the experimental observations in Fig 2, the key thing is that when the animal “adapts” to the expected reward of 0.5 R (not 0.5 u) provided by a predictive cue, it then exhibits identical dopamine responses when presented with reward R, regardless of the value of R.

**We agree with the reviewer and now only refer to 0.5R in the text.**

In Fig 2B, how should we think about the different response with a cue in the context of FCD? This is not discussed in the main text, or I may have missed it.

**We now fixed the text to clarify that in Fig. 2B the cue is identical for all reward magnitudes. In the FCD model this should result in subtractive inhibition by a fixed offset compared to the uncued case, as is observed experimentally. This is in contrast to the case in Fig. 2DEF, where different cues were used for each predicted reward magnitude, which result in identical dopamine responses upon reward presentation.**

Line 228, the authors suddenly refer to runs, without having explained that the motion is assumed to be run-and-tumble-like. Related to above, if dopamine affects run duration, then the model for \chi will not simply be \mu/d0*D, and \beta won’t equal \mu/d0.

**We fixed this mistake in the text, which now states that movement speed (rather than run duration) is proportional to *d*. We also note that the values of** $\boldsymbol{\chi,D}$ **(and thus** $\boldsymbol{\beta}$**) are unchanged for the model variant where *d*** **controls run duration, as we show in Supplementary Information 2, so this modelling choice does not affect our conclusions.**

Line 301: is it true that runs are short compared to the adaptation time for animals? The adaptation time seems quite short in Fig 2. Are there other conditions under which this drift-diffusion model is accurate?

**Unfortunately we are not aware of accurate estimates for run durations in animals in the context of matching experiments, and how they compare with adaptation time (which is on the order of hundreds of milliseconds). Longer run durations would result in a smaller** $\boldsymbol{\beta}$ **(see e.g. derivations in Salek et al. Nat. Comms. 2019), which would in fact bring the estimate of** $\boldsymbol{\beta}$ **closer to the experimentally-observed undermatching – we now added this to the discussion (Lines 456-465):**

***The stochastic model is sufficient for explaining matching behavior, and provides an accurate mechanistic estimate for the matching constant*** $\boldsymbol{\beta}$***. The estimate is derived under mild mechanistic assumptions – that movement speed (or run length) is controlled proportionally by dopamine levels, and that run times are relatively short compared with adaptation time. Improved experimental characterization of movement control by dopamine will allow us to relax these assumptions to get a better estimate for*** $\boldsymbol{\beta}$***. For example, a nonlinear gain for movement speed regulation by dopamine*** $\boldsymbol{v\propto}\boldsymbol{d}^{\boldsymbol{h}}$ ***would result in multiplication by a constant prefactor*** $\boldsymbol{\beta}\boldsymbol{=h\mu/}\boldsymbol{d}_{\boldsymbol{0}}$***, while longer run times would result in a proportional decrease in*** $\boldsymbol{\beta}$ ***(101). As long as these effects are mild, we expect our estimate*** $\boldsymbol{\beta\approx1}$ ***to hold. Importantly, we still expect the matching constant*** $\boldsymbol{\beta}$ ***to be proportional to*** $\boldsymbol{\mu}$ ***and inversely proportional to*** $\boldsymbol{d}_{\boldsymbol{0}}$***.***

**Other conditions, mentioned in the text, are the mean-field assumption on the GABAergic levels, the assumptions mentioned previously on dopaminergic regulation of movement, and other explicit assumptions (such as fixed reorientation frequency).**

In Fig 3B, is the constant offset explained by the FCD model? This is missing in the simulation. Also in Fig 3, how did the authors choose b, the decay length scale of the reward field? What sets this in experiments?

**We now explain the constant offset (also known as the “bias” term) and its explanation in the model in the revised text. This term typically varies between experiments, and is thought to represent a preference (or bias) of the animal to one reward over the other, even when reinforcement rates are equal (Lines 267-275):**

***The matching law, in its time-allocation form (70), posits that the long-term average of the relative amount of time the animal chooses each reward location*** $\boldsymbol{P}\left( \boldsymbol{x}_{\boldsymbol{1}} \right)\boldsymbol{,P}\left( \boldsymbol{x}_{\boldsymbol{2}} \right)$ ***goes as a power*** $\boldsymbol{\beta}$ ***of the ratio of rewards harvested from the locations*** $\boldsymbol{R}_{\boldsymbol{1}}\boldsymbol{,}\boldsymbol{R}_{\boldsymbol{2}}$ ***(Figure 3A,B):***

$$\frac{\boldsymbol{P}\left( \boldsymbol{x}_{\boldsymbol{1}} \right)}{\boldsymbol{P}\left( \boldsymbol{x}_{\boldsymbol{2}} \right)}\boldsymbol{=}{\boldsymbol{k}\left( \frac{\boldsymbol{R}_{\boldsymbol{1}}}{\boldsymbol{R}_{\boldsymbol{2}}} \right)}^{\boldsymbol{\beta}}\boldsymbol{[}4\boldsymbol{]}$$

***Where*** $\boldsymbol{R}_{\boldsymbol{1}}\boldsymbol{,}\boldsymbol{R}_{\boldsymbol{2}}$ ***effectively correspond to the expected reward at each location (the product of rate and amount of reinforcement at each location (70)). The parameter*** $\boldsymbol{k}$ ***is a bias term which corresponds to the tendency of the animal to prefer one reward over another even when reinforcement is equal (***$\boldsymbol{R}_{\boldsymbol{1}}\boldsymbol{=}\boldsymbol{R}_{\boldsymbol{2}}\boldsymbol{)}$***. The bias term typically varies between experiments.***

**Bias is consistent with our model (Lines 338-346):**

***The steady-state distribution can be readily solved, using standard methods of statistical physics, similar to a Boltzmann distribution in a potential field. The motion samples a probability distribution P(x) proportional to a power*** $\boldsymbol{\beta}$ ***of the expected-reward distribution:***

$$\boldsymbol{P}\left( \boldsymbol{x} \right)\boldsymbol{\propto}\boldsymbol{e}^{\boldsymbol{\beta}\log\boldsymbol{R(x)}}\boldsymbol{=R}\left( \boldsymbol{x} \right)^{\boldsymbol{\beta}}\boldsymbol{[}6\boldsymbol{]}$$

***where the power-law*** $\boldsymbol{\beta}$ ***equals the normalized gain of the dopaminergic neurons:*** $\boldsymbol{\beta=}\frac{\boldsymbol{\chi}}{\boldsymbol{D}}\boldsymbol{=}\boldsymbol{\mu}\boldsymbol{d}_{\boldsymbol{0}}^{\boldsymbol{-1}}$***. From Eq.*** $6$ ***we infer that for any two expected rewards*** $\boldsymbol{R}_{\boldsymbol{1}}\boldsymbol{,}\boldsymbol{R}_{\boldsymbol{2}}$ ***the response rates*** $\boldsymbol{P}\left( \boldsymbol{x}_{\boldsymbol{1}} \right)\boldsymbol{,P(}\boldsymbol{x}_{\boldsymbol{2}}\boldsymbol{)}$ ***obey the general matching law presented in Eq.*** $4$***. We note that this results in matching with*** $\boldsymbol{k=1}$***, but any biases in reward preference, which are multiplicative in*** $\boldsymbol{R}_{\boldsymbol{1}}$ ***or*** $\boldsymbol{R}_{\boldsymbol{2}}\boldsymbol{,}$ ***will result in a fixed bias term*** $\boldsymbol{k\neq1}$***.***

**Regarding the decay length-scale, the length-scale that we chose corresponds to a length-scale where convergence to steady-state in simulations occurs relatively rapidly. Other choices length-scale result in identical matching behavior; however, the length-scale affects the rate at which the animal switches between options. We therefore expect that animals that switch more rapidly between options have a longer decay length-scale, while animals that more rarely switch may have a short decay length-scale.**

Lines 415-417: Can the authors explain this claim? The variance of r increases with Y. Why would models based on std normalization not change as Y increases?

**We now clarified in the text that in the examples we keep Y fixed but increase X (and thus keep the variance constant).**

Minor

Dashed lines in Fig 2D are not explained.

**We now explain the dashed lines in the figure legend.**

Typo around line 270 “an underlying choice processes”

**We now fixed this in the text.**

Line 435: Keller and Segel showed that \beta>1 is required for collective migration, as well.

**We thank the reviewer for pointing this out – we now mention this in the text.**

Line 413: 5.5 should be 0.5? It would be helpful here to explicitly spell out in parentheses the values of X and Y in these examples.

**There was a typo in the text that we fixed – 5.5 should have been 11.5. We also explicitly wrote the values of X,Y in the examples as suggested.**

Line 454: Do eye movements really diffuse?

**Random walk models are standard for studying eye movements - we now added references for this to the relevant discussion section. However, like in the case of animal movements, it is likely that these models are only appropriate under certain circumstances.**

Reviewer #2: The authors present a theoretical paper on a possible scheme for the dopamine-reward system. By positing that the system responds to the logarithm of the reward and has the property of fold change detection, they show they can account for some of the properties of the system, namely its rescaling features. They then couple the dynamics of dopamine to movement and show that a proper (simple) coupling (reward-taxis) can lead to accounting for the matching law of operant behavior, i.e. the time spent in various alternatives is proportional to a power $\beta$ of the corresponding rewards. Overall, the paper essentially does a transfer of knowledge from two disparate and far fields, bacterial chemotaxis and dopamine-driven behavior. This is valuable. The paper remains speculative at this stage, which is not necessarily negative but I would suggest the author try to do a better job at the following. Some alternative hypotheses have been put forward in the literature for many of the various features. It would be useful if the authors spent some more time thinking about possible experiments that can be performed to test their various hypotheses and discriminating them from the existing ones. Ideally, the authors could create a well-visible paragraph in the final discussion section where the various experiments are detailed so that experimentalists can pick them up. There is already some of that but I would invite the authors to do more and better.

**We thank the reviewer for the endorsement of our work. We now added a paragraph to the discussion which details experiments to test the most important model predictions (Lines 466-477):**

***The model thus makes experimentally testable predictions on dopaminergic dynamics and their relation to behavioral regulation. The FCD property can be tested by measuring dopaminergic responses to rewards drawn from various reward distributions; we expect identical responses when the distributions are identical up to a scaling constant. As mentioned previously, FCD can also be discriminated from scaling by standard deviation by testing whether responses diminish when the mean of the distribution increases. At the behavioral level, the model predicts that matching is an emergent property of the modulation of movement statistics by dopamine, so that the matching constant*** $\boldsymbol{\beta}$ ***scales proportionally to*** $\boldsymbol{\mu}$ ***and inversely proportional to*** $\boldsymbol{d}_{\boldsymbol{0}}$***, but does not depend on movement parameters such as movement speed, or on any other circuit parameter. This can be tested by performing pharmacological or genetic interventions that affect the relevant parameters, and then testing their effect on matching behavior.***

Two minor misprints:
line 86: an spatial input -> a spatial input
line 87: to higher to dopamine -> to higher dopamine

**We now fixed these in the text.**

Reviewer #3: The authors propose a new way to account for dopaminergic activity in reward seeking setting. To do so they introduce a logarithm in the computations of the reward prediction error signal. This modification results in an ability to explain puzzling experimental data related to scale-invariance, as well as the well documented matching law. It also raises a number of thought-provoking parallels with bacterial chemotaxis.

The work is very well written, very interesting and of major interest for the animal reinforcement learning community.

**We thank the reviewer for this endorsement of our work.**

The discussion proposes very interesting predictions, easily testable.

In the literature linking dopamine with reinforcement learning, a trend has emerged to highlight the possible involvement of dopamine in the regulation of exploration (Frank et al., 2009 ; Eisenegger et al., 2014 ; Costa et al., 2014 ; Cinotti et al., 2019), in addition to its supposed role in learning. This hasn't been discussed in the present paper, and I would be grateful to the authors if they could add a paragraph to their discussion to comment on what their model has to say on that topic, that is closely related to sampling. I think they may have an elegant unifying explanation for these reports.

**We thank the reviewer for these references. As suggested, we now added to the discussion a paragraph discussing the relevance of our work to recent work on the relation between dopamine and exploration (Lines 535-544):**

***The relation between the dopaminergic system and sampling, and in particular the relation between dopaminergic parameters and the inverse temperature parameter*** $\boldsymbol{\beta}$***, may be relevant to recent findings on dopamine and exploration (128, 129). In mice and humans, dopaminergic antagonists appear to specifically increase random exploration, rather than affect learning (128, 129). Under our modelling framework, this effect may correspond to a decrease in*** $\boldsymbol{\beta}$ ***by the treatment, for example, due to a reduction in the effective dopaminergic gain. This would result in altered behavioral output, without necessarily affecting learning. More generally, the sampling framework can provide a quantitative theoretical framework to model the relation between dopamine and various aspects of stochastic exploration, such as novelty-driven exploration (130, 131).***

Minor remarks:
* The authors mention (lines 40-46) the role of dopamine in the invigoration of movement and motivation, but do not cite a very influencial paper on that topic: (Berridge 2007)

* the authors also mention the diversity of dopamine neuron firing patterns (lines 95-97 and line 400), and should probably cite here the early (Matsumoto & Hikosaka, 2009) article.

**We thank the reviewer for pointing us to these citations, which we now refer to in the text.**

* When reporting about Tobler's experimental results (lines 161-174), the authors say that the response to the stimuli increases with expected reward magnitude, while it is not anymore the case at reward delivery. Unfortunately Fig. 2 E and F only illustrate this activity at reward time (right column), but not at stimuli time (the left columns is the activity at reward time without cues). Would it be possible to add this to the figure?
* I am not sure all interested readers will know the concept of advection that appears line 295. Could they add a sentence to explain it, so as to ease the reading for non-physicists?

**We now added an explanation in the text.**

* line 319, an equation number is missing.

**We now fixed this in the text.**

* Line 382, "a simple and strategy" -> "a simple strategy"

**We now fixed this in the text.**

* The authors mention links with hippocampal replay (lines 439-449), as well as the cooperation of multiple decision-making systems (lines 457-463) and may therefore be interested in reading (Cazé et al., 2018). I am not requiring it to be cited in the current paper, but this may be of interest for you.

**We thank the reviewer for bringing this very interesting manuscript to our attention.**

* Could the authors export their supplementary information file in pdf? I could open the provided docx with "Pages", but the equations then have formatting problems.

**We now exported the SI in pdf format.**

References:
Berridge, K. C. (2007). The debate over dopamine’s role in reward: the case for incentive salience. Psychopharmacology, 191(3), 391-431.
Cazé, R., Khamassi, M., Aubin, L., & Girard, B. (2018). Hippocampal replays under the scrutiny of reinforcement learning models. Journal of neurophysiology, 120(6), 2877-2896.
Cinotti, F. et al. Dopamine blockade impairs the exploration-exploitation trade-off in rats. Scientific Reports 9, 1-14 (2019).
Costa, V. D., Tran, V. L., Turchi, J. & Averbeck, B. B. Dopamine modulates novelty seeking behavior during decision making. Behav. Neurosci. 128(5), 556–566 (2014).
Eisenegger, C. et al. Role of dopamine D2 receptors in human reinforcement learning. Neuropsychopharmacology 39(10), 2366–75 (2014).
Frank, M. J., Doll, B. B., Oas-Terpstra, J. & Moreno, F. The neurogenetics of exploration and exploitation: Prefrontal and striatal dopaminergic components, In. Nature Neuroscience 12(8), 1062–1068 (2009).
Matsumoto, M. & Hikosaka, O. Two types of dopamine neuron distinctly convey positive and negative motivational signals. Nature. 459(7248), 837–841 (2009).
