# Supplementary material for: The dopamine circuit as a reward-taxis navigation system
Source: PLoS Comput Biol. 2022 Jul 25;18(7):e1010340. doi: 10.1371/journal.pcbi.1010340 (PMC9352198; doi:10.1371/journal.pcbi.1010340)
Supplement: Attachment — Submitted filename: rebuttal_.docx [file pcbi.1010340.s007.docx]

**Dear Editors,**

**Thank you for the positive consideration of our manuscript and for the referee reports. We apologize for the confusion and the mix up of the versions. We now updated the final version in accordance to the referee comments.**

**Yours,**

**Omer Karin and Uri Alon**

Reviewer #3: I am not sure the authors submitted the latest version of their revisions:

Indeed, I suggested citing Matsumoto & Hikosaka 2009 concerning the variety of the dopaminergic neurons activity, beyond computing RPEs. The authors wrote they now refer to it in the text, but I could not find the reference. Maybe they decided this reference is not relevant, but then please state it clearly in your answer.

**We now added the reference to the text (line 110) as suggested by the referee.**

I also suggested modifying fig 2, but got no answer on that point (maybe the authors would prefer not modify it, but, again, please be explicit).

**We apologize for this, in fact the data for the dopamine response following the cue was available as a supplementary figure, however this was not clear from the text. We now edited both the main text (line 184) and the supplementary figure legend (now Supplementary Figure 2) to clarify this.**

Finally I suggested explaining the term advection, that may not be known by all readers, the authors wrote that an explanation was added to the text, but I could not find it: please could you specify where it is (or add it if it was forgotten).

**We now clarified the meaning of advection in the text (line 315):**

*The advection term corresponds to the average flow at location x.*
