# Supplementary material for: The dopamine circuit as a reward-taxis navigation system
Source: PLoS Comput Biol. 2022 Jul 25;18(7):e1010340. doi: 10.1371/journal.pcbi.1010340 (PMC9352198; doi:10.1371/journal.pcbi.1010340)
Supplement: Peer review history

# Peer review: Decision Letter 0

**DOI:** 10.1371/journal.pcbi.1010340.r001

**Authors:** Thomas Serre, Blake A Richards

20 May 2022

Dear Mr Karin,

Thank you very much for submitting your manuscript "The dopamine circuit as a reward-taxis navigation system" for consideration at PLOS Computational Biology. As with all papers reviewed by the journal, your manuscript was reviewed by members of the editorial board and by several independent reviewers. The reviewers appreciated the attention to an important topic. Based on the reviews, we are likely to accept this manuscript for publication, providing that you modify the manuscript according to the review recommendations. Given the reviewers' comments, we suspect most of the required changes can be addressed through rewriting.

Please prepare and submit your revised manuscript within 30 days. If you anticipate any delay, please let us know the expected resubmission date by replying to this email.

When you are ready to resubmit, please upload the following:

[1] A letter containing a detailed list of your responses to all review comments, and a description of the changes you have made in the manuscript. Please note while forming your response, if your article is accepted, you may have the opportunity to make the peer review history publicly available. The record will include editor decision letters (with reviews) and your responses to reviewer comments. If eligible, we will contact you to opt in or out

[2] Two versions of the revised manuscript: one with either highlights or tracked changes denoting where the text has been changed; the other a clean version (uploaded as the manuscript file).

Important additional instructions are given below your reviewer comments.

Thank you again for your submission to our journal. We hope that our editorial process has been constructive so far, and we welcome your feedback at any time. Please don't hesitate to contact us if you have any questions or comments.

Sincerely,

Blake A Richards

Associate Editor

PLOS Computational Biology

Thomas Serre

Deputy Editor

PLOS Computational Biology

***********************

A link appears below if there are any accompanying review attachments. If you believe any reviews to be missing, please contact ploscompbiol@plos.org immediately:

[LINK]

Given the reviewers' comments, we suspect most of the required changes can be addressed through rewriting.

Reviewer's Responses to Questions

**Comments to the Authors:**

**Please note here if the review is uploaded as an attachment.**

Reviewer #1: In this paper, the authors show that modeling dopamine release as a response to fold changes in expected reward, together with changes in organism motion with dopaminergic neuron firing rates, can produce “reward-taxis” and explain several experimental observations. These include: how the maximum change in neuron firing rates with changes in reward size (Fig 2B), response rescaling when a cue is provided ahead of a reward (Fig 2DE), and matching laws of operant behavior (Fig 3). The authors draw an intriguing analogy to bacterial chemotaxis.

I find the idea interesting and compelling, but I have some comments.

My main comment is that a logical error is made around line 318 and after: while I agree that \\beta should equal \\chi/D, the functional form of \\chi is highly dependent on the specific ways in which dopamine responses alter animal behavior. Therefore, \\beta = \\mu/d0 is only true if one assumes the authors’ simple model for how dopamine elicits changes in behavior, and one cannot in general estimate \\beta from \\mu and d0. You need to know the details of the system to determine an expression for \\chi and thus \\beta. The coincidental agreement between \\beta and \\mu/do in one system (around Line 323) is therefore not convincing.

Some explanations throughout could be clearer. Line 121, it would be helpful to explicitly indicate which quantities depend on time. The explanations around Line 184 are confusing because they refer to both R and u, but R is just a linear function of u. To explain the experimental observations in Fig 2, the key thing is that when the animal “adapts” to the expected reward of 0.5 R (not 0.5 u) provided by a predictive cue, it then exhibits identical dopamine responses when presented with reward R, regardless of the value of R.

In Fig 2B, how should we think about the different response with a cue in the context of FCD? This is not discussed in the main text, or I may have missed it.

Line 228, the authors suddenly refer to runs, without having explained that the motion is assumed to be run-and-tumble-like. Related to above, if dopamine affects run duration, then the model for \\chi will not simply be \\mu/d0*D, and \\beta won’t equal \\mu/d0.

Line 301: is it true that runs are short compared to the adaptation time for animals? The adaptation time seems quite short in Fig 2. Are there other conditions under which this drift-diffusion model is accurate?

In Fig 3B, is the constant offset explained by the FCD model? This is missing in the simulation. Also in Fig 3, how did the authors choose b, the decay length scale of the reward field? What sets this in experiments?

Lines 415-417: Can the authors explain this claim? The variance of r increases with Y. Why would models based on std normalization not change as Y increases?

Minor

Dashed lines in Fig 2D are not explained.

Typo around line 270 “an underlying choice processes”

Line 435: Keller and Segel showed that \\beta>1 is required for collective migration, as well.

Line 413: 5.5 should be 0.5? It would be helpful here to explicitly spell out in parentheses the values of X and Y in these examples.

Line 454: Do eye movements really diffuse?

Reviewer #2: The authors present a theoretical paper on a possible scheme for the dopamine-reward system. By positing that the system responds to the logarithm of the reward and has the property of fold change detection, they show they can account for some of the properties of the system, namely its rescaling features. They then couple the dynamics of dopamine to movement and show that a proper (simple) coupling (reward-taxis) can lead to accounting for the matching law of operant behavior, i.e. the time spent in various alternatives is proportional to a power $\\beta$ of the corresponding rewards. Overall, the paper essentially does a transfer of knowledge from two disparate and far fields, bacterial chemotaxis and dopamine-driven behavior. This is valuable. The paper remains speculative at this stage, which is not necessarily negative but I would suggest the author try to do a better job at the following. Some alternative hypotheses have been put forward in the literature for many of the various features. It would be useful if the authors spent some more time thinking about possible experiments that can be performed to test their various hypotheses and discriminating them from the existing ones. Ideally, the authors could create a well-visible paragraph in the final discussion section where the various experiments are detailed so that experimentalists can pick them up. There is already some of that but I would invite the authors to do more and better.

Two minor misprints:

line 86: an spatial input -> a spatial input

line 87: to higher to dopamine -> to higher dopamine

Reviewer #3: The authors propose a new way to account for dopaminergic activity in reward seeking setting. To do so they introduce a logarithm in the computations of the reward prediction error signal. This modification results in an ability to explain puzzling experimental data related to scale-invariance, as well as the well documented matching law. It also raises a number of thought-provoking parallels with bacterial chemotaxis.

The work is very well written, very interesting and of major interest for the animal reinforcement learning community.

The discussion proposes very interesting predictions, easily testable.

In the literature linking dopamine with reinforcement learning, a trend has emerged to highlight the possible involvement of dopamine in the regulation of exploration (Frank et al., 2009 ; Eisenegger et al., 2014 ; Costa et al., 2014 ; Cinotti et al., 2019), in addition to its supposed role in learning. This hasn't been discussed in the present paper, and I would be grateful to the authors if they could add a paragraph to their discussion to comment on what their model has to say on that topic, that is closely related to sampling. I think they may have an elegant unifying explanation for these reports.

Minor remarks:

* The authors mention (lines 40-46) the role of dopamine in the invigoration of movement and motivation, but do not cite a very influencial paper on that topic: (Berridge 2007)

* the authors also mention the diversity of dopamine neuron firing patterns (lines 95-97 and line 400), and should probably cite here the early (Matsumoto & Hikosaka, 2009) article.

* When reporting about Tobler's experimental results (lines 161-174), the authors say that the response to the stimuli increases with expected reward magnitude, while it is not anymore the case at reward delivery. Unfortunately Fig. 2 E and F only illustrate this activity at reward time (right column), but not at stimuli time (the left columns is the activity at reward time without cues). Would it be possible to add this to the figure?

* I am not sure all interested readers will know the concept of advection that appears line 295. Could they add a sentence to explain it, so as to ease the reading for non-physicists?

* line 319, an equation number is missing.

* Line 382, "a simple and strategy" -> "a simple strategy"

* The authors mention links with hippocampal replay (lines 439-449), as well as the cooperation of multiple decision-making systems (lines 457-463) and may therefore be interested in reading (Cazé et al., 2018). I am not requiring it to be cited in the current paper, but this may be of interest for you.

* Could the authors export their supplementary information file in pdf? I could open the provided docx with "Pages", but the equations then have formatting problems.

References:

Berridge, K. C. (2007). The debate over dopamine’s role in reward: the case for incentive salience. Psychopharmacology, 191(3), 391-431.

Cazé, R., Khamassi, M., Aubin, L., & Girard, B. (2018). Hippocampal replays under the scrutiny of reinforcement learning models. Journal of neurophysiology, 120(6), 2877-2896.

Cinotti, F. et al. Dopamine blockade impairs the exploration-exploitation trade-off in rats. Scientific Reports 9, 1-14 (2019).

Costa, V. D., Tran, V. L., Turchi, J. & Averbeck, B. B. Dopamine modulates novelty seeking behavior during decision making. Behav. Neurosci. 128(5), 556–566 (2014).

Eisenegger, C. et al. Role of dopamine D2 receptors in human reinforcement learning. Neuropsychopharmacology 39(10), 2366–75 (2014).

Frank, M. J., Doll, B. B., Oas-Terpstra, J. & Moreno, F. The neurogenetics of exploration and exploitation: Prefrontal and striatal dopaminergic components, In. Nature Neuroscience 12(8), 1062–1068 (2009).

Matsumoto, M. & Hikosaka, O. Two types of dopamine neuron distinctly convey positive and negative motivational signals. Nature. 459(7248), 837–841 (2009).

**********

**Have the authors made all data and (if applicable) computational code underlying the findings in their manuscript fully available?**

The PLOS Data policy requires authors to make all data and code underlying the findings described in their manuscript fully available without restriction, with rare exception (please refer to the Data Availability Statement in the manuscript PDF file). The data and code should be provided as part of the manuscript or its supporting information, or deposited to a public repository. For example, in addition to summary statistics, the data points behind means, medians and variance measures should be available. If there are restrictions on publicly sharing data or code —e.g. participant privacy or use of data from a third party—those must be specified.

Reviewer #1: Yes

Reviewer #2: None

Reviewer #3: Yes

**********

PLOS authors have the option to publish the peer review history of their article (what does this mean?). If published, this will include your full peer review and any attached files.

If you choose “no”, your identity will remain anonymous but your review may still be made public.

**Do you want your identity to be public for this peer review?** For information about this choice, including consent withdrawal, please see our Privacy Policy.

Reviewer #1: No

Reviewer #2: No

Reviewer #3: **Yes: **Benoît Girard

Figure Files:

While revising your submission, please upload your figure files to the Preflight Analysis and Conversion Engine (PACE) digital diagnostic tool, https://pacev2.apexcovantage.com. PACE helps ensure that figures meet PLOS requirements. To use PACE, you must first register as a user. Then, login and navigate to the UPLOAD tab, where you will find detailed instructions on how to use the tool. If you encounter any issues or have any questions when using PACE, please email us at figures@plos.org.

Data Requirements:

Please note that, as a condition of publication, PLOS' data policy requires that you make available all data used to draw the conclusions outlined in your manuscript. Data must be deposited in an appropriate repository, included within the body of the manuscript, or uploaded as supporting information. This includes all numerical values that were used to generate graphs, histograms etc.. For an example in PLOS Biology see here: http://www.plosbiology.org/article/info%3Adoi%2F10.1371%2Fjournal.pbio.1001908#s5.

Reproducibility:

To enhance the reproducibility of your results, we recommend that you deposit your laboratory protocols in protocols.io, where a protocol can be assigned its own identifier (DOI) such that it can be cited independently in the future. Additionally, PLOS ONE offers an option to publish peer-reviewed clinical study protocols. Read more information on sharing protocols at https://plos.org/protocols?utm_medium=editorial-email&utm_source=authorletters&utm_campaign=protocols

References:

Review your reference list to ensure that it is complete and correct. If you have cited papers that have been retracted, please include the rationale for doing so in the manuscript text, or remove these references and replace them with relevant current references. Any changes to the reference list should be mentioned in the rebuttal letter that accompanies your revised manuscript.

*If you need to cite a retracted article, indicate the article’s retracted status in the References list and also include a citation and full reference for the retraction notice.*

# Author comment: Author response to Decision Letter 0

**DOI:** 10.1371/journal.pcbi.1010340.r002

7 Jun 2022

Attachment Submitted filename: rebuttal_.docx Click here for additional data file.

# Peer review: Decision Letter 1

**DOI:** 10.1371/journal.pcbi.1010340.r003

**Authors:** Thomas Serre, Blake A Richards

23 Jun 2022

Dear Mr Karin,

Thank you very much for submitting your manuscript "The dopamine circuit as a reward-taxis navigation system" for consideration at PLOS Computational Biology. As with all papers reviewed by the journal, your manuscript was reviewed by members of the editorial board and by several independent reviewers. The reviewers appreciated the attention to an important topic. Based on the reviews, we are likely to accept this manuscript for publication, providing that you modify the manuscript according to the review recommendations.

It appears that some of the revisions requested by reviewer 3 did not actually make their way into the submitted text. Please be sure to submit a revised version with these changes.

Please prepare and submit your revised manuscript within 30 days. If you anticipate any delay, please let us know the expected resubmission date by replying to this email.

When you are ready to resubmit, please upload the following:

[1] A letter containing a detailed list of your responses to all review comments, and a description of the changes you have made in the manuscript. Please note while forming your response, if your article is accepted, you may have the opportunity to make the peer review history publicly available. The record will include editor decision letters (with reviews) and your responses to reviewer comments. If eligible, we will contact you to opt in or out

[2] Two versions of the revised manuscript: one with either highlights or tracked changes denoting where the text has been changed; the other a clean version (uploaded as the manuscript file).

Important additional instructions are given below your reviewer comments.

Thank you again for your submission to our journal. We hope that our editorial process has been constructive so far, and we welcome your feedback at any time. Please don't hesitate to contact us if you have any questions or comments.

Sincerely,

Blake A Richards

Associate Editor

PLOS Computational Biology

Thomas Serre

Deputy Editor

PLOS Computational Biology

***********************

A link appears below if there are any accompanying review attachments. If you believe any reviews to be missing, please contact ploscompbiol@plos.org immediately:

[LINK]

It appears that some of the revisions requested by reviewer 3 did not actually make their way into the submitted text. Please be sure to submit a revised version with these changes.

Reviewer's Responses to Questions

**Comments to the Authors:**

**Please note here if the review is uploaded as an attachment.**

Reviewer #1: The authors have addressed my concerns.

Reviewer #3: I am not sure the authors submitted the latest version of their revisions:

Indeed, I suggested citing Matsumoto & Hikosaka 2009 concerning the variety of the dopaminergic neurons activity, beyond computing RPEs. The authors wrote they now refer to it in the text, but I could not find the reference. Maybe they decided this reference is not relevant, but then please state it clearly in your answer.

I also suggested modifying fig 2, but got no answer on that point (maybe the authors would prefer not modify it, but, again, please be explicit).

Finally I suggested explaining the term advection, that may not be known by all readers, the authors wrote that an explanation was added to the text, but I could not find it: please could you specify where it is (or add it if it was forgotten).

**********

**Have the authors made all data and (if applicable) computational code underlying the findings in their manuscript fully available?**

The PLOS Data policy requires authors to make all data and code underlying the findings described in their manuscript fully available without restriction, with rare exception (please refer to the Data Availability Statement in the manuscript PDF file). The data and code should be provided as part of the manuscript or its supporting information, or deposited to a public repository. For example, in addition to summary statistics, the data points behind means, medians and variance measures should be available. If there are restrictions on publicly sharing data or code —e.g. participant privacy or use of data from a third party—those must be specified.

Reviewer #1: Yes

Reviewer #3: Yes

**********

PLOS authors have the option to publish the peer review history of their article (what does this mean?). If published, this will include your full peer review and any attached files.

If you choose “no”, your identity will remain anonymous but your review may still be made public.

**Do you want your identity to be public for this peer review?** For information about this choice, including consent withdrawal, please see our Privacy Policy.

Reviewer #1: No

Reviewer #3: **Yes: **Benoît Girard

Figure Files:

While revising your submission, please upload your figure files to the Preflight Analysis and Conversion Engine (PACE) digital diagnostic tool, https://pacev2.apexcovantage.com. PACE helps ensure that figures meet PLOS requirements. To use PACE, you must first register as a user. Then, login and navigate to the UPLOAD tab, where you will find detailed instructions on how to use the tool. If you encounter any issues or have any questions when using PACE, please email us at figures@plos.org.

Data Requirements:

Please note that, as a condition of publication, PLOS' data policy requires that you make available all data used to draw the conclusions outlined in your manuscript. Data must be deposited in an appropriate repository, included within the body of the manuscript, or uploaded as supporting information. This includes all numerical values that were used to generate graphs, histograms etc.. For an example in PLOS Biology see here: http://www.plosbiology.org/article/info%3Adoi%2F10.1371%2Fjournal.pbio.1001908#s5.

Reproducibility:

To enhance the reproducibility of your results, we recommend that you deposit your laboratory protocols in protocols.io, where a protocol can be assigned its own identifier (DOI) such that it can be cited independently in the future. Additionally, PLOS ONE offers an option to publish peer-reviewed clinical study protocols. Read more information on sharing protocols at https://plos.org/protocols?utm_medium=editorial-email&utm_source=authorletters&utm_campaign=protocols

References:

Review your reference list to ensure that it is complete and correct. If you have cited papers that have been retracted, please include the rationale for doing so in the manuscript text, or remove these references and replace them with relevant current references. Any changes to the reference list should be mentioned in the rebuttal letter that accompanies your revised manuscript.

*If you need to cite a retracted article, indicate the article’s retracted status in the References list and also include a citation and full reference for the retraction notice.*

# Author comment: Author response to Decision Letter 1

**DOI:** 10.1371/journal.pcbi.1010340.r004

28 Jun 2022

Attachment Submitted filename: rebuttal_.docx Click here for additional data file.

# Editor report: Decision Letter 2

**DOI:** 10.1371/journal.pcbi.1010340.r005

**Authors:** Thomas Serre, Blake A Richards

29 Jun 2022

Dear Mr Karin,

We are pleased to inform you that your manuscript 'The dopamine circuit as a reward-taxis navigation system' has been provisionally accepted for publication in PLOS Computational Biology.

Before your manuscript can be formally accepted you will need to complete some formatting changes, which you will receive in a follow up email. A member of our team will be in touch with a set of requests.

Please note that your manuscript will not be scheduled for publication until you have made the required changes, so a swift response is appreciated.

IMPORTANT: The editorial review process is now complete. PLOS will only permit corrections to spelling, formatting or significant scientific errors from this point onwards. Requests for major changes, or any which affect the scientific understanding of your work, will cause delays to the publication date of your manuscript.

Should you, your institution's press office or the journal office choose to press release your paper, you will automatically be opted out of early publication. We ask that you notify us now if you or your institution is planning to press release the article. All press must be co-ordinated with PLOS.

Thank you again for supporting Open Access publishing; we are looking forward to publishing your work in PLOS Computational Biology. 

Best regards,

Blake A Richards

Associate Editor

PLOS Computational Biology

Thomas Serre

Deputy Editor

PLOS Computational Biology

***********************************************************

# Editor report: Acceptance letter

**DOI:** 10.1371/journal.pcbi.1010340.r006

**Authors:** Thomas Serre, Blake A Richards

19 Jul 2022

PCOMPBIOL-D-22-00492R2 

The dopamine circuit as a reward-taxis navigation system

Dear Dr Karin,

I am pleased to inform you that your manuscript has been formally accepted for publication in PLOS Computational Biology. Your manuscript is now with our production department and you will be notified of the publication date in due course.

The corresponding author will soon be receiving a typeset proof for review, to ensure errors have not been introduced during production. Please review the PDF proof of your manuscript carefully, as this is the last chance to correct any errors. Please note that major changes, or those which affect the scientific understanding of the work, will likely cause delays to the publication date of your manuscript. 

Soon after your final files are uploaded, unless you have opted out, the early version of your manuscript will be published online. The date of the early version will be your article's publication date. The final article will be published to the same URL, and all versions of the paper will be accessible to readers.

Thank you again for supporting PLOS Computational Biology and open-access publishing. We are looking forward to publishing your work! 

With kind regards,

Zsofia Freund

PLOS Computational Biology | Carlyle House, Carlyle Road, Cambridge CB4 3DN | United Kingdom ploscompbiol@plos.org | Phone +44 (0) 1223-442824 | ploscompbiol.org | @PLOSCompBiol
